# Mechanisms of individual variation in large herbivore diets: Roles of spatial heterogeneity and state‐dependent foraging

**DOI:** 10.1002/ecy.3921

**Published:** 2023-01-03

**Authors:** Reena H. Walker, Matthew C. Hutchinson, Arjun B. Potter, Justine A. Becker, Ryan A. Long, Robert M. Pringle

**Affiliations:** ^1^ Department of Fish and Wildlife Sciences University of Idaho Moscow Idaho USA; ^2^ Department of Ecology and Evolutionary Biology Princeton University Princeton New Jersey USA; ^3^ Department of Zoology and Physiology University of Wyoming Laramie Wyoming USA

**Keywords:** dietary niche differentiation, endogenous resources, Gorongosa National Park, Mozambique, intraspecific individual variability, niche variation hypothesis, optimal foraging theory, resource partitioning, state‐dependent behavior, trophic specialization

## Abstract

Many populations of consumers consist of relatively specialized individuals that eat only a subset of the foods consumed by the population at large. Although the ecological significance of individual‐level diet variation is recognized, such variation is difficult to document, and its underlying mechanisms are poorly understood. Optimal foraging theory provides a useful framework for predicting how individuals might select different diets, positing that animals balance the “opportunity cost” of stopping to eat an available food item against the cost of searching for something more nutritious; diet composition should be contingent on the distribution of food, and individual foragers should be more selective when they have greater energy reserves to invest in searching for high‐quality foods. We tested these predicted mechanisms of individual niche differentiation by quantifying environmental (resource heterogeneity) and organismal (nutritional condition) determinants of diet in a widespread browsing antelope (bushbuck, *Tragelaphus sylvaticus*) in an African floodplain‐savanna ecosystem. We quantified individuals' realized dietary niches (taxonomic richness and composition) using DNA metabarcoding of fecal samples collected repeatedly from 15 GPS‐collared animals (range 6–14 samples per individual, median 12). Bushbuck diets were structured by spatial heterogeneity and constrained by individual condition. We observed significant individual‐level partitioning of food plants by bushbuck both within and between two adjacent habitat types (floodplain and woodland). Individuals with home ranges that were closer together and/or had similar vegetation structure (measured using LiDAR) ate more similar diets, supporting the prediction that heterogeneous resource distribution promotes individual differentiation. Individuals in good nutritional condition had significantly narrower diets (fewer plant taxa), searched their home ranges more intensively (intensity‐of‐use index), and had higher‐quality diets (percent digestible protein) than those in poor condition, supporting the prediction that animals with greater endogenous reserves have narrower realized niches because they can invest more time in searching for nutritious foods. Our results support predictions from optimal foraging theory about the energetic basis of individual‐level dietary variation and provide a potentially generalizable framework for understanding how individuals' realized niche width is governed by animal behavior and physiology in heterogeneous landscapes.

## INTRODUCTION

Classic niche theory and most models of trophic interactions assume that individuals in a population are ecologically equivalent (Hutchinson, 1957; MacArthur & Levins, 1967; Voltera, 1926). Yet, many populations consist of relatively specialized individuals that use only a subset of the resources exploited by the population at large (Araújo et al., 2011; Bolnick et al., 2003; Van Valen, 1965). Consistent differences among conspecific individuals can increase a population's resilience to disturbance (Reusch et al., 2005), influence competition and coexistence (Hart et al., 2016), and increase the probability of speciation events (Fryxell & Lundberg, 1994). In strongly interacting species such as large mammalian herbivores, which shape community structure and ecosystem functions (Guy et al., 2021; Pringle et al., 2007; Ripple et al., 2015), individual‐level diet variation may also have system‐wide consequences. To date, however, few studies have investigated the occurrence and/or degree of individual diet variation in ungulates, let alone the mechanisms that produce it (but see, e.g., Bison et al., 2015; Jesmer et al., 2020; Pansu et al., 2019b).

Optimal foraging theory (OFT) provides one framework for predicting how individual herbivores should choose foods (Belovsky, 1978; MacArthur & Pianka, 1966; Owen‐Smith & Novellie, 1982; Stephens & Krebs, 1986). OFT posits that animals should seek to maximize the average rate of energy intake, both in the choice of where to feed (i.e., the marginal value theorem; Charnov, 1976) and in the diet chosen there (i.e., the basic prey model; Emlen, 1966; Stephens & Krebs, 1986), by balancing the “opportunity cost” of stopping to consume an available food item against the cost of moving on to search for something more nutritious. Accordingly, individual diets are predicted to be contingent on encounter rate and the relative availability of profitable food items, which together should determine the amount of time a forager spends searching for preferred foods and whether a food item is accepted or rejected when it is encountered (Charnov, 1976; MacArthur & Pianka, 1966; Westoby, 1974). Thus, in systems where high‐quality foods are heterogeneously distributed, individual diets may be both differentiated and “optimal” depending on the distribution of resources in each individual's home range (Stephens & Krebs, 1986). Despite the prominent role of food distribution in OFT models, few studies have evaluated how landscape heterogeneity influences individual diet variation in wild populations (Araújo et al., 2011).

Experimental studies have shown that conspecifics select different diets even when exposed to identical resources (Parent et al., 2014), indicating that factors other than spatial heterogeneity shape patterns of individual diet variation. State‐dependent behavior—patterns of activity modulated by an individual's underlying physiological state (McNamara & Houston, 1996)—might interact with resource distribution to drive dietary variation. Nutritional condition (i.e., energy reserves available for maintenance, growth, and reproduction; Parker et al., 2009) is a state variable with especially high potential for influencing the behavior of foraging animals (Cook et al., 2013; Long et al., 2014). Animals in poor condition are weakly buffered against starvation, which decreases the opportunity cost of handling low‐quality resources relative to searching for higher‐quality food items (Mathot & Dall, 2013). OFT predicts that foraging animals in poor condition should be less selective and more willing to accept lower‐quality foods in order to maximize caloric yield per time by reducing energy invested in searching (Emlen, 1966; Owen‐Smith et al., 2010; Stephens & Krebs, 1986). Simulations have supported this prediction by showing that optimal model foragers with greater energy reserves invest more time searching for high‐quality food items than those with low energy reserves (Nonacs, 2001). In addition, controlled experiments have shown that satiated individuals choose different prey than those that are less well‐buffered against starvation (Gill, 2003; Perry, 1987). Despite this theoretical foundation, however, we know of no previous study that has explored state‐dependent foraging behavior as a mechanism for generating individual dietary variation among free‐ranging large herbivores.

We studied the interaction between environmental (resource heterogeneity) and organismal (nutritional condition) determinants of diet composition in bushbuck (*Tragelaphus sylvaticus*), an ~45‐kg African bovid. We longitudinally sampled bushbuck diets in Mozambique's Gorongosa National Park by collecting multiple fecal samples from 15 Global Positioning System (GPS)‐collared individuals (6 to 14 samples per animal collected over 42–56 days). We analyzed these samples using DNA metabarcoding, enabling taxonomically precise measurement of diet composition and richness at the individual level (Kartzinel et al., 2015, 2019; Pansu et al., 2019b; Pringle & Hutchinson, 2020). Bushbuck in Gorongosa show high fidelity to small (generally <3‐km^2^) home ranges distributed across two broad habitat types (Atkins et al., 2019; Daskin et al., 2022), which allowed us to evaluate the role of spatial resource heterogeneity at different scales (population level and partitioned by habitat affiliation) in generating individual diet differentiation. Further, bushbuck are nonseasonal breeders, leading to wide variation among individuals in reproductive status and associated nutritional condition (owing to the high costs of gestation and lactation; Cook et al., 2013). This life‐history trait allowed us to evaluate the role of state‐dependent foraging behavior in shaping individual diets.

We hypothesized that diet selection is constrained by spatial variation in the distribution of food plants because foragers with small home ranges, high site fidelity, and limited mobility have only a subset of the population‐level resource base available to them. Based on this hypothesis, we predicted (a) that individuals consistently eat distinct diets (i.e., each individual uses a small fraction of the food plants used by the population at large) and (b) that dietary dissimilarity between individuals increases as a function of the distance and vegetation dissimilarity between home ranges. We further hypothesized that state‐dependent foraging behavior is a key mechanism generating variation in diet selection among conspecific individuals, because tradeoffs faced by consumers (e.g., forage intake vs. search time) are modulated by nutritional condition. Accordingly, we predicted that bushbuck in better condition have less variable diets, because they invest more time searching for the best food items and thus (a) accept fewer food types (leading to lower dietary richness), (b) search their home ranges more exhaustively (higher intensity‐of‐use index), and (c) have diets that are higher quality on average (higher percentage digestible protein [DP]) than individuals in poor condition.

## METHODS

### Study site and species

**FIGURE 1:**
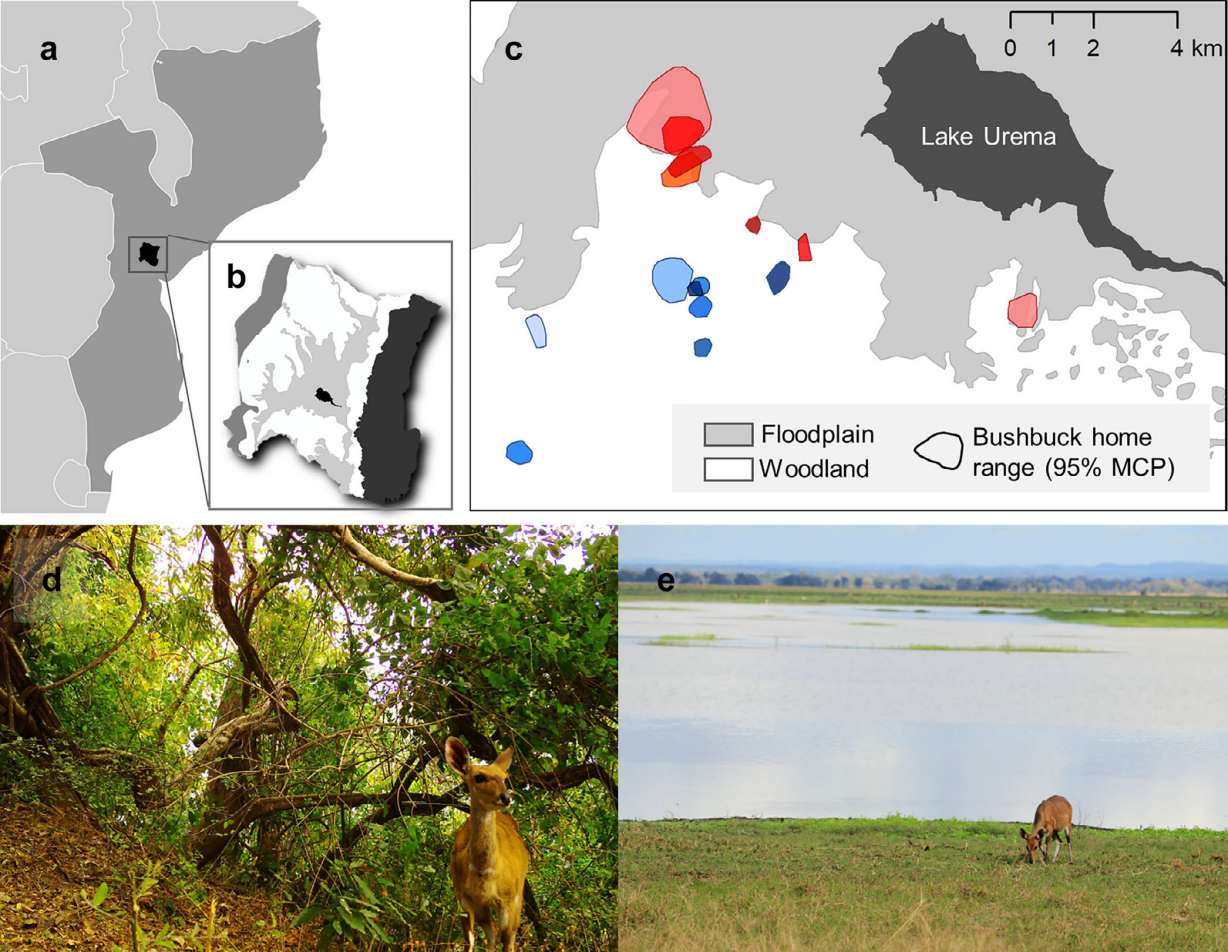
Bushbuck habitat affiliations in Gorongosa National Park, Mozambique. (a) Gorongosa is located in central Mozambique and (b) consists of four major habitat zones (from left: western escarpment [medium gray], woodland [white], floodplain [light gray], and eastern escarpment [dark gray], as well as Lake Urema [black]). (c) Bushbuck home ranges (95% minimum convex polygons [MCPs]) within our study area; blue polygons (different shades indicate different individuals) represent individual home ranges that lie only within woodland habitat, whereas red polygons represent individual home ranges that overlap with floodplain habitat. (d) Female bushbuck foraging in typical woodland habitat; photo credit: Reena H. Walker. (e) Female bushbuck foraging in typical floodplain habitat; photo credit: Matthew C. Hutchinson.

Gorongosa is situated at the southern end of the Great Rift Valley (−18.97, 34.35; Figure 1a,b). Our study site at the southern end of the park comprises two main habitat types: savanna woodland and floodplain grassland (Figure 1c; Atkins et al., 2019; Becker et al., 2021; Stalmans et al., 2019). These habitats have very different plant communities (Figure 1d,e; Appendix S1: Figure S1). The woodland is a mix of *Acacia*, *Combretum*, and palm savanna dotted with termitaria thickets; the floodplain is an open and comparatively homogeneous landscape of grasses, forbs, and subshrubs (Stalmans & Beilfuss, 2008). The majority of annual rainfall (700–900 mm; Stalmans et al., 2019) occurs during the wet season (November–April), and primary productivity peaks during this period. Food and water become increasingly limited as the dry season advances. We quantified bushbuck diets during the mid‐dry seasons (June–August) of 2018 and 2019.

Bushbuck are spiral‐horned antelopes (Bovidae: Bovinae: Tragelaphini) that occur widely across sub‐Saharan Africa. They are solitary but not aggressively antisocial, such that home ranges overlap and individuals often forage in close proximity (Estes, 1991). As strict browsers, bushbuck feed almost exclusively on trees, shrubs, and forbs to the exclusion of grasses (Daskin et al., 2022; Kartzinel & Pringle, 2020; Pansu et al., 2019b, 2022; Potter et al., 2022). Bushbuck also use woody plants for concealment and are considered “dependent on thick cover” for predator avoidance (Kingdon, 1997, p. 352). In Gorongosa, however, bushbuck have increasingly occupied the Lake Urema floodplain over the last 20 years; in that period, large‐mammal populations were recovering from >90% declines during the Mozambican Civil War (1977–1992), and predation risk was low, enabling bushbuck to expand into the treeless floodplain (Atkins et al., 2019). By 2018, ungulate biomass in Gorongosa had recovered to nearly prewar levels, with large populations of midsize browsing antelopes, including 1700 bushbuck, 1900 nyala (*Tragelaphus angasii*), 1900 kudu (*Tragelaphus strepsiceros*), and 6000 impala (*Aepyceros melampus*) (Stalmans et al., 2019). Lions (*Panthera leo*) were the dominant top carnivore (≥100 individuals), but bushbuck were not among their recorded prey (Bouley et al., 2018). African wild dogs (*Lycaon pictus*) and leopards (*Panthera pardus*) were extirpated during the war, but a pack of 14 wild dogs was reintroduced in June 2018 (the start of our study) followed by another pack of 15 in December 2019, and these animals have fed heavily on bushbuck (Bouley et al., 2021); at least one leopard was also present in the area by 2018. Thus, Gorongosa's large‐mammal fauna was abundant and nearly intact at the time of our study, but species' relative abundances remained skewed and predation pressure low (but increasing) relative to the prewar baseline.

### Animal handling and condition measurements

In June 2018 and 2019, we captured adult female (*n* = 20) and male (*n* = 10) bushbuck as part of the long‐term Allometry of Spiral‐Horned Antelopes: Movement Ecology & Diet (ASHAMED) study. We chemically immobilized bushbuck via remote injection (1.5–2.5 mg thiafentanil, 50–60 mg ketamine, 10–15 mg azaperone) from a vehicle. Darts were equipped with radiotransmitters to facilitate recovery of darted individuals. We fitted each individual with an iridium satellite GPS collar (VERTEX Lite, Vectronic Aerospace) programmed to record hourly locations and equipped with a very‐high‐frequency transmitter that we used to relocate collared individuals for diet sampling via radio telemetry. GPS collars were remotely triggered to drop off 1 year after deployment.

We collected a fecal sample (>5 pellets) from the rectum of each immobilized bushbuck for molecular analysis (see DNA metabarcoding). For female bushbuck, we used an ultrasound (Ibex Pro, EI Medical Imaging) to confirm the presence of a fetus and palpated the udder to determine lactation status (lactating or not lactating). For each individual, we collected body measurements (weight, body and hind‐foot length, chest girth), ultrasonography data (maximum rump‐fat depth, thickness of biceps femoris and longissimus dorsi muscles), and palpation scores at the sacrosciatic ligament, lumbar vertebrae, sacrum, base of the tail, and caudal vertebrae (based on protocols developed for North American ungulates; Cook et al., 2010; Stephenson et al., 2020). Because equations for converting these measurements to an estimate of ingesta‐free body fat have not been validated for African ungulates, we followed Atkins et al. (2019) and Becker et al. (2021) in using principal component analysis to develop an index of relative nutritional condition (Appendix S2). All animal‐handling procedures were approved by the Animal Care and Use Committees of the University of Idaho (No. IACUC‐2019‐32) and Princeton University (No. 2075F‐16) and followed guidelines established by the American Society of Mammologists (Sikes & The Animal Care and Use Committee of the American Society of Mammologists, 2016).

### Habitat and space‐use analyses

We used 95% minimum convex polygons (MCPs) derived from a subset of the hourly GPS‐location data to estimate individual bushbuck home ranges during the diet‐sampling period in each year (June–August; Appendix S3: Table S1). We used two complementary approaches to assess the role of spatial heterogeneity in structuring diet composition. First, following Tobler's (1970) first law of geography (“everything is related to everything else, but near things are more related than distant things”), we calculated the distance between each pair of bushbuck home‐range centroids (the arithmetic mean position of GPS fixes from each individual) as a proxy for similarity of the vegetation communities available to bushbuck. We lacked home‐range‐specific measurements of plant community composition, but we verified that plant community dissimilarity increased with geographic distance, both between and within habitats (Appendix S4: Figure S1). Second, we quantified the dissimilarity of vertical vegetation structure between each pair of bushbuck home ranges using airborne light detection and ranging (LiDAR) data collected in August 2019 (Wooding Geospatial Solutions Ltd.). LiDAR flights were conducted at 880 m above ground level at 110 knots; the resulting terrain and vegetation elevation measurements had a horizontal spatial resolution of 0.5 m and a vertical resolution of 0.1 m. For each home range, we calculated the proportion of LiDAR points classified as ground (0 m), low vegetation (<0.3 m), medium vegetation (0.3–1.6 m), and high vegetation (>1.6 m), following guidelines of the American Society for Photogrammetry and Remote Sensing (ASPRS, 2008). We then calculated the Bray–Curtis index of compositional dissimilarity in vegetation structure (proportion of LiDAR points classified as ground, low, medium, and high) between each pair of bushbuck home ranges.

We quantified differences in searching behavior by bushbuck within their home ranges using the intensity‐of‐use index (Almeida et al., 2010; Hailey & Coulson, 1996; Loretto & Vieira, 2005). We calculated intensity of use as the ratio of total movement distance to the square of the area of movement (100% MCP) during the diet sampling period for each bushbuck (Appendix S3: Table S1) using the “intensity_use” function in the amt package (Singer et al., 2019) in R version 3.5.3 (R Core Team, 2018). Bushbuck sampled in 2019 had hourly GPS data only during the first 21 days after capture, owing to a change in sampling rate required for a separate research project; thus, we restricted all GPS data to 21 days after capture to avoid bias due to unequal sample sizes. Results using restricted versus full data sets were qualitatively equivalent (Appendix S5: Figure S1). Although this index does not distinguish among active behaviors (e.g., cannot distinguish movements related to searching for food versus seeking shade), intensity of use is proportional to the time spent active per unit of area and increases when animals (i) follow highly tortuous paths, (ii) move slowly, or (iii) perform search loops (Almeida et al., 2010). Thus, the index quantifies how active bushbuck are within their home ranges, which enabled us to test the prediction that bushbuck in good condition spend more time searching than do individuals in poor condition (see *Statistical analyses*).

### DNA metabarcoding

We quantified bushbuck diet composition using fecal DNA metabarcoding following protocols that we previously used to study herbivore diets in Gorongosa (Atkins et al., 2019; Becker et al., 2021; Branco et al., 2019; Guyton et al., 2020; Pansu et al., 2019b; Potter et al., 2022). After collecting an initial fecal sample at the time of capture, we used radio telemetry to relocate each collared bushbuck every 1–2 days. Upon relocation we confirmed individual identity via an ID number written on the collar belting and then observed individuals for up to 2 h from distances of 5–100 m (depending on the density of vegetation and wariness of the animal). Bushbuck typically returned to a nonvigilant state (eyes not fixated on the observer and ears relaxed while resting or foraging) after <10 min of observation. We observed individuals until they defecated, whereupon we recorded their distance from the observer and the nearest landmarks to the defecation site. We then searched that area for the fresh fecal sample, selected >5 debris‐free fecal pellets using nitrile gloves and deposited them in an unused plastic zip bag. We stored samples on ice for <6 h before preprocessing them in Gorongosa's laboratory as follows. We homogenized each fecal sample and transferred a pea‐sized subsample into a labeled tube containing silica beads and buffer (Xpedition Stabilization/Lysis Solution, Zymo Research Corporation). We vortexed samples to lyse cells and froze them at −20°C pending transport to Princeton University, where we stored them at −80°C. Prior to transport, we subjected each sample to an antiviral heat treatment (72°C for 30 min) as mandated by the US Department of Agriculture (Permit 130123 to Robert M. Pringle).

DNA extraction and PCR were conducted in a Biosafety Level 2 facility dedicated to fecal DNA analysis, with physically separated pre‐ and post‐PCR rooms and laminar flow hoods for PCR preparation. We extracted DNA from each sample in a biosafety cabinet using Zymo Xpedition Soil/Fecal MiniPrep kits (as per manufacturer protocols) in batches of 29 samples and one negative extraction control. In triplicate PCR replicates of each sample, we amplified the P6 loop of the chloroplast *trn*L(UAA) intron, a standard region for metabarcoding degraded plant DNA, using primers with a unique 8‐nt tag at the 5′ end that enabled pooling of uniquely identifiable PCR products for sequencing in a single high‐throughput run (Taberlet et al., 2007).

A detailed description of the laboratory and bioinformatic protocols used to analyze our samples is in Guyton et al. (2020). Briefly, sequencing was performed on an Illumina HiSeq 2500, and data were processed using the OBITools pipeline (Boyer et al., 2016). Sequences of low quality (low alignment score, unexpected barcode length, ambiguous nucleotides, singletons) were discarded; the remaining sequences were considered molecular operational taxonomic units (mOTUs, “taxa”) and identified by primary comparison to a local plant DNA reference library collected in Gorongosa (Pansu et al., 2019a) and by secondary comparison to a global database compiled from the European Molecular Biology Laboratory (release 134) only if the local‐library assignment score was <98%. After filtering, we averaged the number of reads across all retained PCR replicates for each sample and removed mOTUs accounting for <1% of reads per sample (Guyton et al., 2020). We rarefied sample read depth to 7000 to facilitate comparisons among samples. From these data, we determined the presence/absence and relative read abundance (RRA) of each plant taxon in each sample, which we used to quantify individual dietary richness and variation (Walker et al., 2022). Results in the main text are based on RRA, which is an informative proxy for the proportional consumption of plant taxa in comparative analyses of large‐herbivore diets using the *trn*L‐P6 marker (Craine et al., 2015; Kartzinel et al., 2015; Willerslev et al., 2014) and generally provides a more robust portrait of diet composition than occurrence‐based data (Deagle et al., 2019) because the latter inflate the importance of uncommon food items (which in turn account for the majority of foods in vertebrate diets; Hutchinson et al., 2022). Although RRA is subject to several sources of error that can influence the proportional representation of particular plant taxa (e.g., amplification bias, differential digestion, variation in chloroplast density) and is thus an imperfect reflection of true biomass consumption, any systematic biases should be consistent across the samples analyzed here, and idiosyncratic biases should be mitigated by our standardized pipeline and quality‐control steps (Deagle et al., 2019). As a further sensitivity check, we also present results based on presence/absence data, which yielded similar inferences about the generality of individual niche differences and their dependence on spatial heterogeneity (Appendix S6: Table S1, Figures S1–S4).

We were unable to obtain a fecal sample from every individual during every observation period, which resulted in unequal sample sizes across individuals. We limited our analyses to individuals for which we obtained ≥6 samples in the 2 months after collaring (Appendix S3: Table S1). For analyses of individual variation and dietary richness (which require multiple samples per individual and may be sensitive to which samples are included for each individual; see *Statistical analyses*), we rarified to six samples per individual and performed the statistical test in each of 1000 resampling iterations (bootstrapping). For other analyses that require just one measure of diet per individual (see *Statistical analyses*), we calculated “standardized diets” for each individual as the mean RRA of each mOTU across 1000 bootstrapping iterations.

### Diet quality

Dry‐season DP content of foliage from 204 of Gorongosa's most common plant species was quantified as part of a concurrent study (Potter et al., 2022). We estimated the quality of individual bushbuck diets by calculating the weighted average of DP in the standardized diet of each bushbuck, using the RRA of each mOTU as the weighting factor (Atkins et al., 2019; Becker et al., 2021; Branco et al., 2019; Walker et al., 2022). Although this approach is subject to the previously listed caveats about the quantitative interpretation of RRA (see *DNA metabarcoding*), we verified our RRA‐based results with occurrence (presence/absence) data by calculating the weighted average of DP with each plant mOTU weighted equally (Appendix S6: Figure S3). Plant taxa for which nutrient data were available accounted for a median of 98% of standardized bushbuck diets (range 83%–100%).

### Statistical analyses

We tested each of our predictions at two scales: the population level and partitioned by habitat affiliation (floodplain or woodland). Following Atkins et al. (2019), we classified bushbuck as “floodplain‐associated” if their home range (95% MCP) overlapped with the treeless floodplain grassland during the sampling period (Figure 1; Appendix S3: Table S1). Floodplain‐associated bushbuck (*n* = 7) spent an average of 40% (range 6%–71%) of their time in this treeless interior of the floodplain, differentiating them from bushbuck that spent 0% of their time in the floodplain grassland (“woodland‐associated” bushbuck; *n =* 8).

We calculated the Bray–Curtis index of compositional dissimilarity between each pair of fecal samples and ordinated the values using nonmetric multidimensional scaling (NMDS) to visualize patterns of dietary dissimilarity both within and among individual bushbuck (Kartzinel et al., 2015; Pansu et al., 2019b). To test our prediction that individuals would consistently eat distinct diets, we conducted permutational multivariate analysis of variance (perMANOVA) on the Bray–Curtis distance matrix for each pair of fecal samples, running 9999 permutations with year (2018 or 2019) as a blocking factor to control for any year effects and individual ID as the main predictor (“adonis2” in the vegan package in R; Oksanen et al., 2020). To test for pairwise differences among bushbuck diets, we conducted post hoc tests using a Holm–Bonferroni adjustment to control for the familywise error rate both across and within habitats (“pairwise.adonis” in the funfuns package in R; Holm, 1979; Trachsel, 2021).

To test our prediction that bushbuck occupying home ranges with similar forage availability (proxied by distance between home‐range centroids; Appendix S4: Figure S1) and vegetation structure would consume similar diets, we used Mantel tests (Pansu et al., 2019b) to evaluate relationships between pairwise interindividual dissimilarity of standardized diets (Bray–Curtis distance) and (i) pairwise geographic distance between home‐range centroids and (ii) pairwise dissimilarity of vegetation structure (Bray–Curtis distance).

We used linear regression to evaluate the relationships between nutritional condition and intensity of use, percentage DP, and dietary richness to test our prediction that bushbuck in better nutritional condition invest more time searching for high‐quality foods and thus have less variable diets than bushbuck in poorer condition. We calculated population‐level dietary richness as the mean number of mOTUs present in all standardized bushbuck diets across bootstrapping iterations. We calculated individual dietary richness as the mean number of mOTUs in each bushbuck's standardized diet across bootstrapping iterations. To evaluate the influence of nutritional condition on dietary richness relative to other factors, we fit competing multiple regression models that included nutritional condition, reproductive state (lactating female, nonlactating female, or male), and habitat affiliation (woodland or floodplain) as candidate predictors. We ranked models using Akiake's information criterion for small sample size (AIC_c_) and evaluated relative model performance by calculating Akaike weights (AIC_*ŵ*_) (Burnham & Anderson, 2002). To assess the importance of longitudinal sampling for accurately characterizing individual diets, we (i) reran all analyses using one randomly drawn sample from the full suite of samples collected from each individual to estimate dietary richness (averaged over *n* = 1000 random trials), (ii) compared these estimates with those from standardized longitudinal bootstrapping (*n* = 6 per individual) using Welch's two‐sample *t‐*tests, and (iii) plotted species‐accumulation curves (“spaccum” function in the vegan package in R; Oksanen et al., 2020) to evaluate how the cumulative number of mOTUs present in a bushbuck diet varied as a function of sample size using all samples per individual (Colwell & Coddington, 1994; Soberon & Llorente, 1993).

## RESULTS

### Role of spatial heterogeneity in individual diet variation

Population‐level dietary richness was 95.6 ± 3.34 taxa (mean ± SD across 1000 resampling iterations), whereas individual‐level dietary richness ranged from 12.8 ± 1.44 to 31.1 ± 3.40 taxa (mean ± SD among all individuals = 21.2 ± 5.81). Thus, individual diets comprised 13%–33% of the total plant taxa used by the population.

**FIGURE 2:**
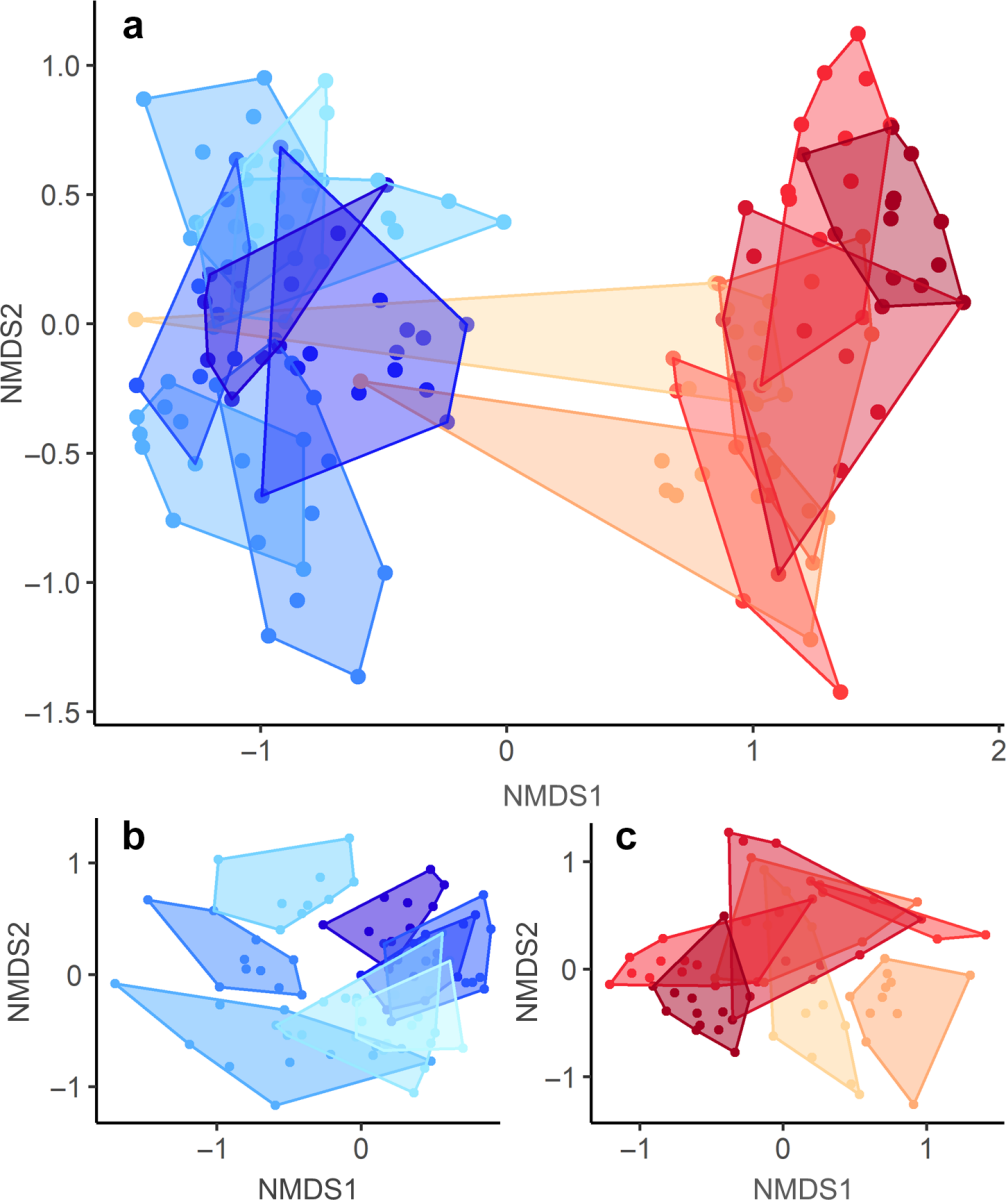
Nonmetric multidimensional scaling (NMDS) ordinations showing relative dissimilarity in taxonomic composition of individual fecal samples (points; *n* = 160) and dry‐season diets (polygons; *n* = 15) of bushbuck in Gorongosa. Results (a) for full population and (b, c) partitioned by habitat association (woodland in blue and floodplain in red). Points in closer proximity to one another indicate more similar diets; polygons are convex hulls around all samples from each individual. Two individuals captured in woodland habitat moved into the floodplain shortly after collaring, where outlying red points in (a) are those collected at capture; the initial woodland samples from these individuals were excluded from our analysis of floodplain diets in (c). We observed significant differences among individual diets both at the population level (perMANOVA: pseudo‐*F*_14,144_ = 15.21, *p* < 0.001, *R* ^2^ = 0.60) and within habitat types (woodland: pseudo‐*F*_7,80_ = 9.97, *p* < 0.001, *R* ^2^ = 0.47; floodplain: pseudo‐*F*_6,62_ = 8.39, *p* < 0.001, *R* ^2^ = 0.45).

After controlling for year effects, we observed marked differences in diet composition among individuals both at the population level (perMANOVA: pseudo‐*F*_14,144_ = 15.21, *p* < 0.001, *R* ^2^ = 0.60) and within habitat types (woodland: pseudo‐*F*_7,80_ = 9.97, *p* < 0.001, *R* ^2^ = 0.47; floodplain: pseudo‐*F*_6,62_ = 8.39, *p* < 0.001, *R* ^2^ = 0.45); 96% of 105 pairwise contrasts between individuals were statistically significant after controlling for multiple comparisons (Appendix S3: Table S2). These differences are clear in NMDS ordinations of all samples collected per individual (Figure 2). Notably, we observed strong shifts in the diets of two females that moved from woodland into the floodplain immediately after capture: The sample collected at capture from each of these individuals reflects a woodland‐type diet whereas the rest are characteristic of floodplain‐affiliated animals (Figure 2a). The two initial samples from these individuals were excluded from floodplain‐specific diet analyses.

**FIGURE 3:**
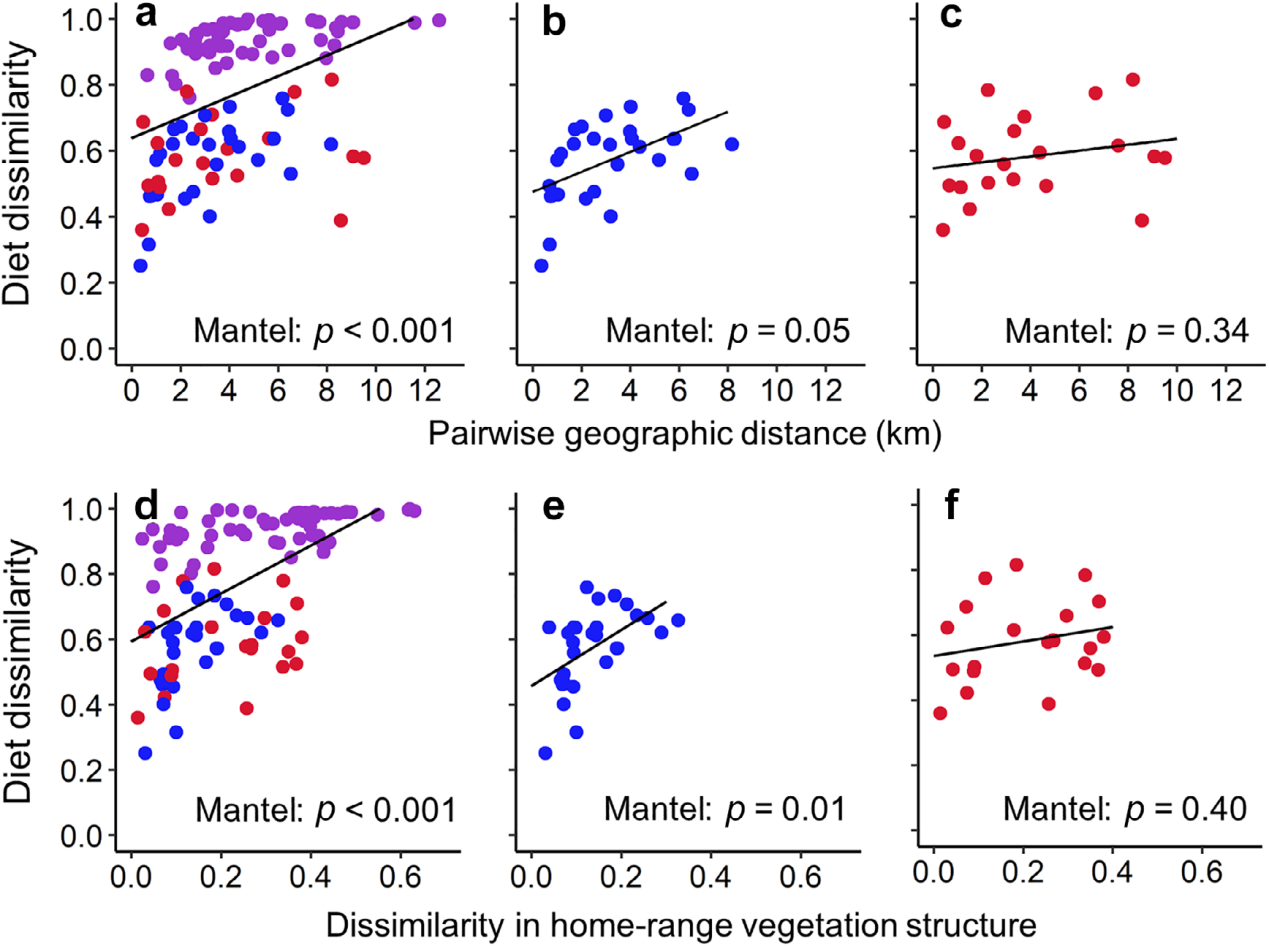
Relationships between bushbuck diet composition and spatial variation in distribution of resources at Gorongosa. We evaluated the relationship between pairwise diet dissimilarity (Bray–Curtis index) and distance between home‐range centroids (km) for (a) all pairs of GPS‐collared bushbuck, (b) bushbuck with home ranges affiliated with woodland habitat, and (c) those affiliated with floodplain habitat. Additionally, we evaluated the relationship between pairwise diet dissimilarity and dissimilarity in home‐range vegetation structure (Bray–Curtis index) between (d) all pairs of GPS‐collared bushbuck, (e) bushbuck with home ranges in woodland habitat, and (f) bushbuck with home ranges in floodplain habitat. Blue points illustrate comparisons between pairs of woodland‐affiliated individuals, red points between pairs of floodplain‐affiliated individuals, and purple points between woodland‐ and floodplain‐affiliated individuals. We quantified vegetation structure by calculating the proportion of LiDAR points classified as ground‐level, low, medium, and high vegetation, and by using the Bray–Curtis index to quantify pairwise compositional dissimilarity between home ranges based on those proportions. In each panel, *p‐*values are from Mantel's permutation tests for similarity between two matrices.

The location and vegetation structure of bushbuck home ranges strongly influenced diet composition. Geographic distance between home‐range centroids, our validated proxy for plant community dissimilarity (Appendix S4: Figure S1), was positively related to dietary dissimilarity between individuals at the population level (Mantel test, *p* < 0.001; Figure 3a) and within woodland habitat (Figure 3b) but not within the floodplain (Figure 3c). Similarly, dissimilarity in vegetation structure between home ranges was positively related to dietary dissimilarity at the population level and in woodlands (Mantel test, *p* < 0.001; Figure 3d,e) but not in the floodplain (Figure 3f). Analyses based on occurrence‐based dietary data in lieu of RRA gave similar results (Appendix S6: Figure S2).

### Role of state‐dependent foraging in individual diet variation

**FIGURE 4:**
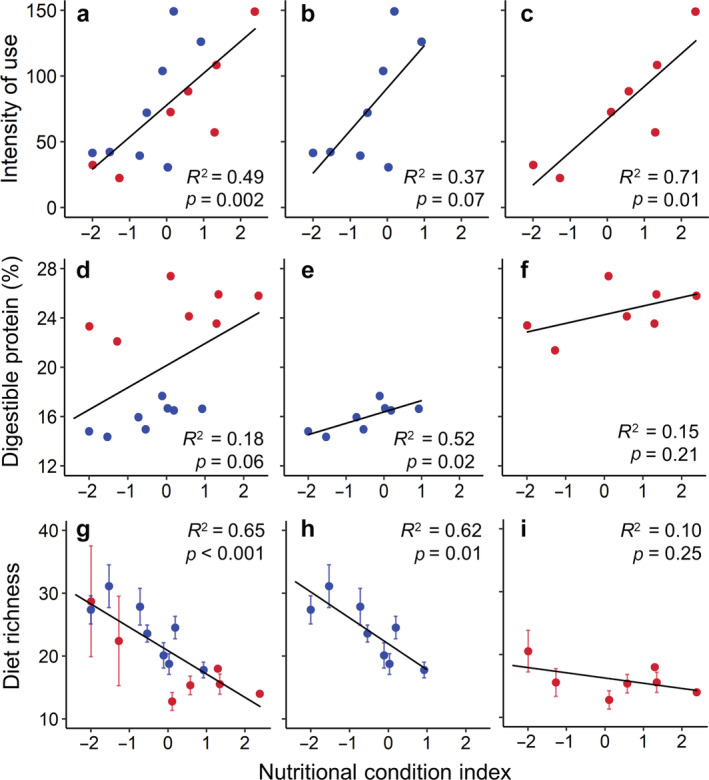
Relationships between a multivariate index of nutritional condition (see *Methods*) and bushbuck foraging behaviors in Gorongosa. Blue points represent bushbuck with home ranges affiliated with woodland habitat; red points represent bushbuck with home ranges affiliated with floodplain habitat. Consistent with predictions of optimal foraging theory, individuals in good nutritional condition (a) searched their home ranges more intensively and (d) had higher‐quality and (g) narrower diets (mean ± SD unique molecular operational taxonomic units). These relationships were still evident when we analyzed only (b, e, h) bushbuck that lived in woodland habitat but not for (c, f, i) bushbuck that lived in floodplain habitat. *R* ^2^ and *p*‐values are from ordinary least‐squares linear regression models.

Nutritional condition influenced bushbuck searching behavior, diet quality, and dietary richness. Bushbuck in better condition had higher intensity‐of‐use indices—that is, spent more time actively searching their home ranges per unit area—than those in poorer condition. This correlation was evident at the population level (β_condition_ = 24.3, *p* < 0.001, *R* ^2^ = 0.49; Figure 4a) and within each habitat (woodland: β_condition_ = 32.3, *p* = 0.07, *R* ^2^ = 0.37; floodplain: β_condition_ = 24.9, *p* = 0.01, *R* ^2^ = 0.71; Figure 4b,c), as well as when we used all hourly GPS locations collected during the diet sampling period instead of limiting them to the first 21 days after capture (Appendix S5: Figure S1).

Bushbuck in better condition tended to eat higher‐quality diets (DP: β_condition_ = 1.79, *p* = 0.06, *R* ^2^ = 0.18; Figure 4d). This population‐level trend was driven by the higher average values of nutritional condition and dietary DP in the floodplain (Welch's two‐sample *t*‐test: ${\bar{\mathrm{DP}}}_{\text{floodplain}}$ = 24.5, ${\bar{\mathrm{DP}}}_{\text{woodland}}$ = 15.9, *p* < 0.001). However, nutritional condition was also positively correlated with dietary DP within woodland (β_condition_ = 0.92, *p* = 0.03, *R* ^2^ = 0.52; Figure 4e), whereas we found no correlation within the floodplain (β_condition_ = 0.07, *p* = 0.21, *R* ^2^ = 0.15; Figure 4f).

Bushbuck in better condition had lower dietary richness (narrower realized niches) than those in poorer condition (Figure 4g). Indeed, nutritional condition was the sole predictor variable in the best regression model of dietary richness (β_condition_ = −3.71, *p* < 0.001, *R* ^2^ = 0.65) and was included in all of the top four candidate models (Table 1). This relationship held for bushbuck in woodland habitat (β_condition_ = −4.12, *p* = 0.01, *R* ^2^ = 0.62) but was not observed among bushbuck in the floodplain (β_condition_ = −0.083, *p* = 0.25, *R* ^2^ = 0.10) (Figure 4h,i). Analyzing dietary Shannon diversity in lieu of richness gave qualitatively similar results (Appendix S7: Figure S1).

**TABLE 1:** Competing multiple regression models for explaining variation in individual dietary richness. “Habitat” is a categorical indicator of which habitat type (floodplain or woodland) affiliated with each bushbuck home range. “Lactation” is a categorical indicator of reproductive status (lactating, non‐lactating, or male). We included these covariates to control for variation in diet richness unaccounted for by nutritional condition (see *Methods*).

Model	Adjusted *R* ^2^	ΔAIC_c_	AIC_*ŵ*_
Nutritional condition	0.65	0	0.541
Nutritional condition + habitat	0.70	0.433	0.436
Nutritional condition + lactation	0.63	6.843	0.018
Nutritional condition + habitat + lactation	0.65	10.173	0.003
Habitat	0.21	12.244	0.001
Lactation	0.27	14.807	0.000
Habitat + lactation	0.27	16.940	0.000

Abbreviations: AIC_c_, corrected Akaike information criterion; AIC_*ŵ*_, Akaike weights.

### Role of longitudinal sampling in inferences about individual diet variation

Analyzing a single diet sample per individual underestimated diet breadth: Diets inferred from one sample had <50% of the food‐plant richness estimated from standardized longitudinal sampling (Welch's two‐sample *t*‐test: ${\bar{X}}_{\text{single}}$ = 10, ${\bar{X}}_{\text{longitudinal}}$ = 21, *p* < 0.001). Species‐accumulation curves showed that even 6 or 10 samples were not universally sufficient for individual dietary richness to asymptote (Appendix S3: Figure S3), despite being far greater than the (typically single) individual sample size commonly used in studies of individual diet variation in ungulates (Bison et al., 2015; Jesmer et al., 2020; Pansu et al., 2019b) and other taxa (Araújo et al., 2011).

## DISCUSSION

Although patterns of diet variation and individual specialization have been documented in diverse taxa (Araújo et al., 2011; Bolnick et al., 2003), empirical investigation of the mechanisms that lead to differentiation among individuals is limited. This is especially true for large herbivores, which are often depicted as highly generalized consumers but may in fact eat relatively narrow sets of plant taxa (Hutchinson et al., 2022). By drawing on the ability to repeatedly sample individual diets with high taxonomic resolution, we show that bushbuck eat distinct subsets of the foods used by the population across both large and small spatial scales. We further show that individual differences in dietary richness and composition arise from two complementary mechanisms: spatial heterogeneity in resource distribution and variation in nutritional condition among individuals. These findings are consistent with predictions from OFT about the energetic underpinnings of individual specialization, suggesting a potentially generalizable framework for understanding how individuals' realized dietary niches are constrained by behavior and physiology.

### Spatial heterogeneity structures individual dietary niche

OFT predicts that the opportunity cost of stopping to consume an available food item versus the cost of moving on to search for a more nutritious food item is modulated by the distribution of high‐quality food across the landscape (e.g., marginal value theorem and basic prey choice models; Charnov, 1976; Emlen, 1966; MacArthur & Pianka, 1966). Thus, individual dietary differences should emerge in environments where food types are heterogeneously distributed and each forager's search area is limited. Consistent with these predictions, we observed significant differences among bushbuck diets both at the population level and within habitat types. Much of the individual dietary variation we observed stemmed from differences in the structure and composition of food plants among bushbuck home ranges, as evidenced by both the clear separation of woodland‐ and floodplain‐associated bushbuck diets and the interactive effect of landscape heterogeneity and home‐range location on individual dietary niches. In general, bushbuck that lived closer together (and thus had access to more similar food plants; Appendix S4) and had similar vegetation structure within their home ranges consumed more similar diets.

These trends were not significant for floodplain bushbuck, despite generally pronounced individual niche differences in that habitat. This might at first seem intuitive, given that the treeless floodplain interior is structurally homogeneous relative to woodland (Appendix S1). Yet floodplain bushbuck varied in their use of the savanna at the floodplain edge (Figure 1c), such that the range of structural dissimilarity between floodplain home ranges was as great as that in woodland (Figure 3e,f); moreover, plant community dissimilarity increased with geographic distance within and across habitats (Appendix S4). We hypothesize that the weak influence of distance and structural dissimilarity on diet differentiation in the floodplain stems from the more uniformly high‐quality forage in that habitat (Atkins et al., 2019; Becker et al., 2021), where flooding annually resets succession and replenishes soil nutrients (Tinley, 1977). Floodplain diets were uniformly lower in richness and higher in DP than woodland diets (Figure 4), and the top five floodplain food taxa (*Mimosa pigra*, *Ludwigia adscendens*, *Ambrosia maritima*, *Faidherbia albida*, *Bergia mossambicensis*) collectively accounted for >50% of each individual's standardized diet and were among the most protein‐rich plants in either habitat (Walker et al., 2022). By foraging selectively on a subset of abundant, high‐quality plants in their home ranges, floodplain bushbuck might decouple individual dietary variation from the geographic structure in plant community composition. In this respect, our null results in that habitat would be consistent with OFT because trade‐offs between intake and search time are relaxed in environments with a homogeneous distribution of high‐quality food.

Despite the importance of resource abundance and distribution in theoretical models of prey choice (Stephens & Krebs, 1986) and niche differentiation (Van Valen, 1965), few studies of dietary variation and individual specialization in wild populations explicitly consider the role of resource distribution in driving differences among individuals (Araújo et al., 2011). Consequently, there is little empirical support for the rather intuitive prediction that segregation across habitat types leads to greater diet differentiation among individuals (Araújo et al., 2011; but see: Darimont et al., 2009; Layman et al., 2007). Our results show that the spatial distribution of resources influences diet composition across scales, underscoring the importance of incorporating measures of resource availability into future studies of individual diet differentiation. Further exploration of the role of landscape heterogeneity would benefit from detailed data on food‐plant availability and palatability. However, the latter is extremely difficult to quantify for free‐ranging animals, and our results show that even selectivity (use relative to availability) is hard to measure at the individual level. For example, we have shown that the subpopulation of bushbuck in Gorongosa's floodplain strongly selects for the shrub *M. pigra* (Pansu et al., 2019b), but accurate individual‐level assessment would entail measuring plant availability within each bushbuck's home range.

The effects of landscape heterogeneity on dietary specialization could have important implications for herbivore community dynamics. Population‐wide, bushbuck diets include plant taxa from both woodland and floodplain habitats, which should relax intraspecific competition via niche complementarity (Bolnick et al., 2011) but leads to higher dietary overlap with heterospecific competitors (Pansu et al., 2019b). The impacts of such diffuse interspecific niche overlap on species coexistence and population abundance remain unresolved; theoretically, alternative outcomes are possible. On the one hand, individual variation can destabilize coexistence by weakening between‐species niche differences and compounding the effects of demographic stochasticity; on the other hand, it can ease interspecific competition and dampen population fluctuations across heterogeneous landscapes (Bolnick et al., 2011; Hart et al., 2016; Stump et al., 2022). Evaluating the strength and outcomes of interspecific competition in antelopes remains a formidable challenge (Prins, 2016), and our study highlights the need to incorporate intraspecific niche variation in this effort.

### State‐dependent foraging constrains individual diet variation

OFT models of patch use and prey choice predict that in heterogeneous resource landscapes, individuals with high energy reserves should invest more time searching, be more discriminating, and thus have narrower, higher‐quality diets than individuals that are less well buffered against starvation (Emlen, 1966; Mathot & Dall, 2013). We found that bushbuck in better condition generally searched their home ranges more intensively for less diverse but more nutritious diets than bushbuck in poorer condition. This pattern diverged only in the floodplain, where the tradeoff between search time and food quality is relaxed and the corresponding relationships between nutritional condition, search intensity, and diet quality are therefore expected to dissipate. These results are consistent with OFT (Emlen, 1966; Svanbäck & Bolnick, 2005) and with simulation models of state‐dependent foraging showing that individuals with higher energy reserves search longer for higher‐quality food (Nonacs, 2001). Moreover, experimental work has demonstrated that animals at higher risk of starvation are less “choosy” when selecting among food types or patches (Barnett et al., 2007), suggesting that our findings may be general across diverse taxa.

State‐dependent foraging can result in consistent dietary differences among individuals when positive feedbacks exist between nutritional condition and constraints on forage selection (i.e., individuals in good condition face fewer constraints; Sih et al., 2015). In this “rich get richer” scenario, differences in diet variation between individuals in good versus poor condition should be sustained through time (Bolnick et al., 2003). Such “disruptive” intraspecific resource partitioning, in which some individuals are able to be increasingly specialized while others are forced to remain generalized, is reported less frequently than the scenario in which all individuals are similarly specialized on subsets of the population‐level diet (Araújo et al., 2011; Estes et al., 2003; Vander Zanden et al., 2013; but see Darimont et al., 2007; Jesmer et al., 2020; West, 1986) and may be a potent selective force insofar as differences in condition are correlated with fitness. State‐dependent behavior may thus provide a mechanistic explanation for patterns of nested resource partitioning (Araújo et al., 2010; Carlson et al., 2021) that, under OFT, emerge when individuals share similar food preferences but differ in the degree to which they accept less‐preferred resources (Svanbäck & Bolnick, 2005). We note, however, that nutritional condition is dynamic and varies with factors other than foraging success (e.g., reproductive state), which may disrupt the feedback loop and result in individuals intermittently trading places along a generalist–specialist continuum.

Although we lack the data to explicitly test whether mechanisms other than state‐dependent foraging modulate food preference or diet composition (e.g., inherited or learned preferences; Estes et al., 2003; Taper & Case, 1985), our results are consistent with simulation analyses in which behavioral adjustments to physiological state enhanced fitness in the absence of individual preferences (Nonacs, 2001). Together with evidence that nutritional condition is likely to be “reset” many times during the average lifespan of a bushbuck due to factors other than foraging strategy (Parker et al., 2009), these lines of evidence suggest that the observed relationships among nutritional condition and diet breadth/quality are more likely to reflect animals' behavioral adjustment to their state than to stem from intrinsic preferences that consistently lead to good nutritional condition and a competitive advantage. Parsing the roles of inherited or learned preference versus state‐dependent foraging in generating diet variation among free‐ranging animals represents a fruitful avenue for future research.

### Caveats and considerations

We focused on the roles of spatial heterogeneity and state‐dependent behavior in driving individual variation, but predation risk also modulates these relationships. A study conducted in Gorongosa before the return of wild dogs and leopards in 2018 documented a “landscape of fearlessness” in which bushbuck increasingly occupied the floodplain from 2002 to 2016 (Atkins et al., 2019). In this way, risk relaxation enabled bushbuck to capitalize on the ecological opportunity presented by high‐quality forage in the floodplain, contributing to the broad‐scale pattern of individual variation documented here; the recovery of large carnivores in the park may increasingly limit bushbuck to habitats with more concealment cover and thereby reduce the extent of individual differentiation at the population level. Crucially, however, all of the same patterns observed at the population level also held within the woodland habitat historically preferred by bushbuck (Tinley, 1977), and predation on bushbuck accelerated during our study (Bouley et al., 2021), indicating that our conclusions were not an artifact of predation regime.

Our results are based on just 15 individuals, yet this sample size is commensurate with previous studies investigating mechanisms of diet selection by large mammalian herbivores (e.g., Atkins et al., 2019; Cerling et al., 2006). Moreover, we know of no previous study that quantified and compared large‐herbivore diets with high taxonomic resolution via longitudinal sampling of known individuals. Collecting multiple fecal samples per individual allowed us to characterize individual diets more thoroughly than in previous studies, most of which used single samples of fecal or gut contents to represent individual niche width (e.g., Araújo et al., 2009; Costa et al., 2008; Pansu et al., 2019b; Redjadj et al., 2014). Although analyzing single samples would not have qualitatively altered the conclusions of this study (on the contrary, it would have tended to exaggerate the degree of individual differentiation; Figure 2), the fact that even at least six samples per individual failed to fully capture individual dietary richness is a caveat regarding our quantitative metrics. The potential impacts of overestimating individual dietary differentiation by undersampling depends on the question being asked. For example, some studies of individual dietary variation specify a simple threshold for determining whether a population is composed of specialist or generalist individuals (e.g., Vander Zanden et al., 2013). However, our results support the contention (e.g., Novak & Tinker, 2015) that measures of dietary variation are highly sensitive to the temporal scale and intensity of sampling and that reliance on threshold values may therefore compromise inference when sampling intensity is insufficient to robustly quantify individual‐level diet breadth.

### Conclusions

Foraging decisions are conditioned upon a variety of extrinsic and intrinsic constraints. Empirical evidence increasingly shows that broad‐scale patterns of habitat selection constrain fine‐scale differences in resource use among individuals (e.g., Feiner et al., 2019; L'Hérault et al., 2013; Zerba & Collins, 1992), and theory shows that these differences can scale up to exert strong (albeit variable) effects on interspecific interactions and population dynamics (Bolnick et al., 2011; Hart et al., 2016; Stump et al., 2022). Understanding the mechanistic bases of individual variation is thus crucial to understanding community organization. By drawing on an uncommon wealth of information on landscape structure, animal movement, diet composition, and nutritional condition, we have shown that spatial heterogeneity and state dependence interact with space use to regulate individual variation. Accounting for these factors is now more possible than ever and should lead to rapid progress in understanding both the causes and consequences of individual specialization.

Our study provides empirical support for OFT as a framework for generating and testing hypotheses about the behavioral mechanisms that drive variation in individual realized niche width in the context of energy supply and demand. Given their strong mechanistic underpinnings, we propose that the relationships documented in our study are likely generalizable across an array of taxa and ecosystems. We encourage future tests of this proposition that focus on parsing the relative roles of extrinsic versus intrinsic constraints in determining individual niche width.

## CONFLICT OF INTEREST

The authors declare no conflicts of interest.

## Supporting information


Appendix S1.

Appendix S2.

Appendix S3.

Appendix S4.

Appendix S5.

Appendix S6.

Appendix S7.

## Data Availability

Data sets utilized for this research are available in Dryad as follows: Pansu et al. (2019a), https://doi.org/10.5061/dryad.63tj806; Walker et al. (2022), https://doi.org/10.5061/dryad.crjdfn364.
