# Supplementary material for: Mechanisms of individual variation in large herbivore diets: Roles of spatial heterogeneity and state‐dependent foraging
Source: Ecology. 2023 Jan 3;104(2):e3921. doi: 10.1002/ecy.3921 (PMC10078531; doi:10.1002/ecy.3921)
Supplement: Supplementary file 1 — Appendix S1. [file ECY-104-0-s004.pdf]

Supporting information. Walker, R. H., M. C. Hutchinson, A.B. Potter, J. A. Becker, R. A. Long, and R. M. Pringle. 2022. **Mechanisms of individual variation in large herbivore diets: roles of spatial heterogeneity and state-dependent foraging.** *Ecology*.

Appendix S1. Aerial photographs illustrating woodland and floodplain habitat types in Gorongosa National Park, Mozambique**.**

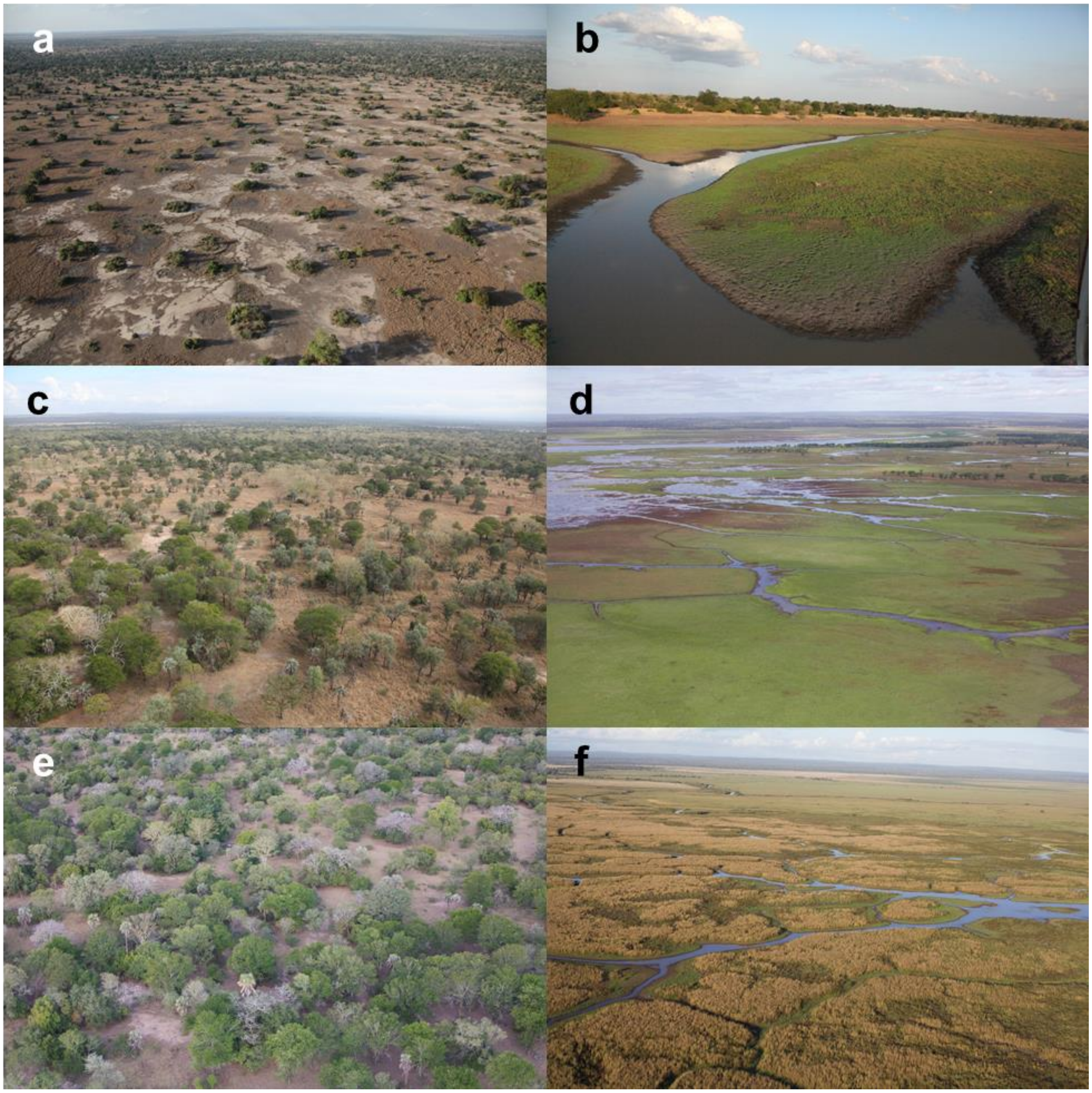

Figure S1. Representative aerial photographs of woodland (A,C,E) and floodplain (B,D,F) habitats in Gorongosa. Woodland habitats include a mix of *Acacia*, *Combretum*, and palm savanna, are commonly dominated by termitaria thickets (A, clusters of trees and shrubs that grow on termite mounds), and span a range of canopy covers from relatively open forest and salt pans (C) to denser, closed-canopy forest (E). The floodplain grassland habitat is a productive (B), seasonally flooded (D) landscape dominated by grasses, forbs, and subshrubs (F) bordered by sparsely wooded savanna (visible in B, D). Photo credits: R.M.P.
