# Supplementary material for: Mechanisms of individual variation in large herbivore diets: Roles of spatial heterogeneity and state‐dependent foraging
Source: Ecology. 2023 Jan 3;104(2):e3921. doi: 10.1002/ecy.3921 (PMC10078531; doi:10.1002/ecy.3921)
Supplement: Supplementary file 2 — Appendix S2. [file ECY-104-0-s007.pdf]

**Supporting information**. Walker, R. H., M. C. Hutchinson, A.B. Potter, J. A. Becker, R. A. Long, and R. M. Pringle. 2022. **Mechanisms of individual variation in large herbivore diets: roles of spatial heterogeneity and state-dependent foraging.** *Ecology*.

**Appendix S2**. Results of principal component analysis used to develop an index of relative nutritional condition.

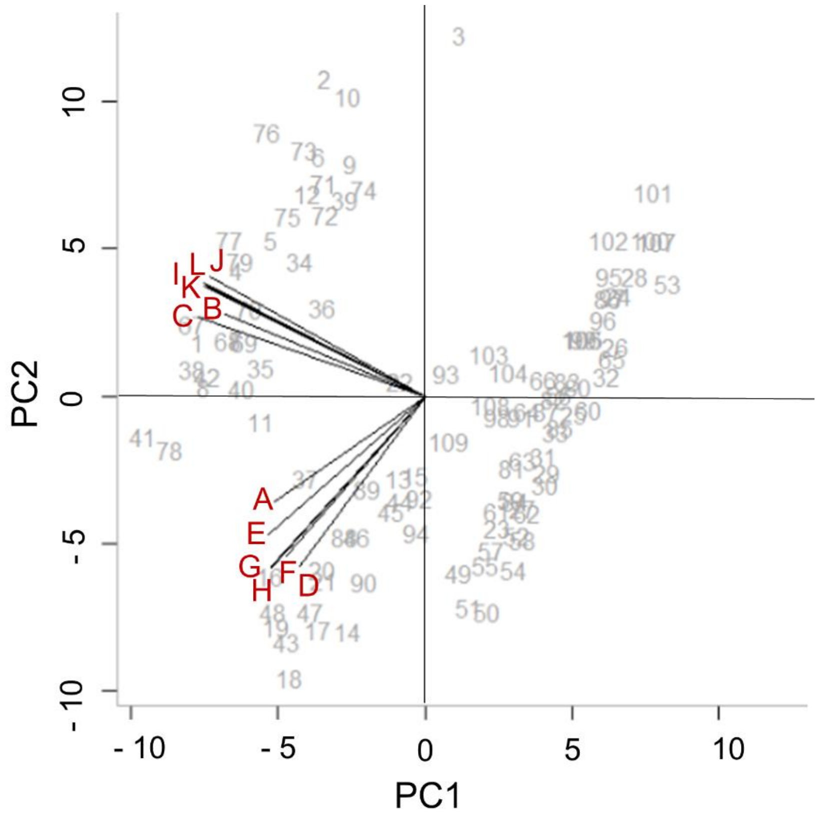


**Figure S1.** Principle component analysis of 12 nutritional condition metrics collected from adult female ($n$ = 112) and male ($n$ = 25) *Tragelaphus* antelopes (bushbuck, nyala, and kudu) in Gorongosa National Park, Mozambique 2014–2019. Nutritional condition metrics were collected at the time of capture and included: (A) maximum rump fat depth, (B) thickness of the *biceps femoris* muscle, (C) thickness of the *longissimus dorsi* muscle, (D) palpation score at the sacrosciatic ligament, (E) palpation score at the lumbar vertebrae, (F) palpation score at the sacrum, (G) palpation score at the base of tail, (H) palpation score at the caudal vertebrae, (I) chest girth, (J) body length, (K) hind foot length, and (L) body weight. Together, Principle Component 1 (PC1) and Principle Component 2 (PC2) explained > 80% of the variance in these data. Black arrows show the projections of the original variables, and each grey number represents an individual antelope. Nutritional condition metrics associated with body size (e.g., muscle thicknesses, body weight) loaded most strongly onto PC1 and those associated with body fat (e.g., palpation scores, max fat) loaded most strongly onto PC2. In ungulates, body fat represents energy stores available for maintenance and reproduction and is commonly used as a measure of individuals' overall nutritional condition. Thus, we used the inverse of PC2 as an intuitive index of nutritional condition of bushbuck so that larger scores reflect individuals in better nutritional condition.

**Table S1.** Summary of principle component analysis of 12 nutritional condition metrics from adult female (*n* = 112) and male (*n* = 25) *Tragelaphus* antelopes in Gorongosa National Park, Mozambique 2014–2019. Each column (PC1-12) represents a principle component. >80% of the variance in these metrics was explained by the first two principle components (54.2% and 27.3% respectively).

| | PC1 | PC2 | PC3 | PC4 | PC5 | PC6 | PC7 | PC8 | PC9 | PC10 | PC11 | PC12 |
|---|---|---|---|---|---|---|---|---|---|---|---|---|
| Standard deviation | 2.550 | 1.808 | 0.805 | 0.649 | 0.580 | 0.538 | 0.480 | 0.357 | 0.310 | 0.207 | 0.175 | 0.086 |
| Proportion of variance explained | 0.542 | 0.273 | 0.054 | 0.035 | 0.028 | 0.024 | 0.019 | 0.011 | 0.008 | 0.004 | 0.003 | 0.001 |
| Cumulative proportion of variance | 0.542 | 0.814 | 0.868 | 0.903 | 0.931 | 0.955 | 0.975 | 0.985 | 0.993 | 0.997 | 0.999 | 1.000 |

**Table S2**. Principle component loadings for each of 12 nutritional condition metrics from adult female (*n* = 112) and male (*n* = 25) *Tragelaphus* antelopes in Gorongosa National Park, Mozambique 2014–2019. Variables associated with body size loaded most strongly onto PC1, whereas variables associated with the amount of body fat loaded most strongly onto PC2.

| | PC1 | PC2 | PC3 | PC4 | PC5 | PC6 | PC7 | PC8 | PC9 | PC10 | PC11 | PC12 |
|---|---|---|---|---|---|---|---|---|---|---|---|---|
| Max_fat | -0.23 | -0.23 | 0.71 | -0.43 | 0.43 | -0.11 | -0.08 | 0.05 | -0.06 | 0.00 | 0.01 | 0.01 |
| B_femoris | -0.31 | 0.18 | 0.25 | 0.15 | -0.14 | 0.80 | -0.22 | 0.04 | 0.26 | 0.06 | 0.03 | 0.03 |
| L_dorsi | -0.35 | 0.17 | -0.06 | 0.01 | -0.02 | 0.10 | -0.04 | -0.52 | -0.74 | 0.01 | -0.13 | 0.04 |
| SS_ligament | -0.19 | -0.37 | 0.38 | 0.41 | -0.43 | -0.12 | 0.53 | -0.15 | 0.06 | -0.06 | 0.00 | -0.01 |
| Lumbar_vert | -0.24 | -0.30 | -0.12 | 0.68 | 0.45 | -0.14 | -0.37 | 0.12 | -0.03 | 0.01 | -0.04 | -0.02 |
| Sacrum | -0.22 | -0.35 | -0.42 | -0.20 | 0.39 | 0.38 | 0.56 | 0.10 | -0.01 | 0.00 | 0.00 | -0.01 |
| Base_tail | -0.24 | -0.37 | -0.14 | -0.23 | -0.48 | -0.02 | -0.27 | 0.52 | -0.26 | 0.19 | -0.24 | 0.02 |
| Caudal_vert | -0.24 | -0.38 | -0.25 | -0.27 | -0.17 | -0.10 | -0.33 | -0.53 | 0.38 | -0.18 | 0.24 | 0.00 |
| Chest_girth | -0.34 | 0.24 | -0.07 | 0.01 | -0.04 | -0.18 | 0.08 | 0.28 | -0.06 | -0.23 | 0.53 | 0.60 |
| Body_length | -0.33 | 0.26 | -0.06 | -0.05 | 0.04 | -0.18 | 0.08 | 0.03 | 0.30 | -0.39 | -0.71 | 0.13 |
| Hind_foot | -0.34 | 0.24 | -0.07 | -0.03 | 0.04 | -0.26 | 0.12 | -0.09 | 0.26 | 0.81 | -0.01 | -0.01 |
| Net_weight | -0.34 | 0.25 | -0.04 | -0.02 | -0.05 | -0.13 | 0.05 | 0.20 | -0.03 | -0.25 | 0.28 | -0.78 |
