# Supplementary material for: Mechanisms of individual variation in large herbivore diets: Roles of spatial heterogeneity and state‐dependent foraging
Source: Ecology. 2023 Jan 3;104(2):e3921. doi: 10.1002/ecy.3921 (PMC10078531; doi:10.1002/ecy.3921)
Supplement: Supplementary file 4 — Appendix S4. [file ECY-104-0-s005.pdf]

**Supporting information**. Walker, R. H., M. C. Hutchinson, A.B. Potter, J. A. Becker, R. A. Long, and R. M. Pringle. 2022. **Mechanisms of individual variation in large herbivore diets: roles of spatial heterogeneity and state-dependent foraging.** *Ecology.*

**Appendix S4.** Supplemental results supporting the assumption that geographic distance is positively related to plant community dissimilarity

We used data from two prior vegetation surveys ('understory plots' in the floodplain and forage transects in the woodland) and two proxies for vegetation community composition within bushbuck home ranges (termite mound density and vegetation structure) to evaluate Tobler's (1970) first law of geography ("everything is related to everything else, but near things are more related than distant things") within our study area. Together, these four avenues of analysis affirm our assumption that geographic distance between home range centroids is a suitable proxy for dissimilarity in plant community composition.

*Understory plots in floodplain plant communities*
We used previously published data (Pansu et al. 2019) from recent vegetation surveys to quantify the relationship between plant community composition and geographic distance within the treeless interior of the floodplain habitat in Gorongosa. Tinley (1977) established 18 1-ha monitoring plots along three parallel transects extending outwards from Lake Urema (Fig. S1a). In June-August 2015, Pansu et al. (2019) resurveryed these plots by randomly placing 1215 1-$m^2$ quadrats and estimating the areal cover of each plant species and amount of bare ground in each quadrat using the Braun-Blanquet (1932) method; each species and bare ground estimate was binned according to its percent cover (1 = <5%; 2 = 6-25%; 3 = 26-50%; 4 = 51-75%; 5 = 76-95%; 6 = 96-100%). Bins were converted into relative-abundance estimates for each species using the median value of each bin (2.5, 15, 37.5, 62.5, 85, 98).

To test our prediction that plots farther away from one another differ more in plant community composition than closer plots, we used a Mantel test to evaluate the relationships between 1) the dissimilarity in the relative-abundance of plant species (Bray-Curtis dissimilarity) and average distance among quadrats within plots, and 2) the dissimilarity in the relative-abundance of plant species (Bray-Curtis distance) and the pairwise geographic distance between plots. We observed a significant, positive relationship between plant community dissimilarity and pairwise geographic distance within and among plots (Fig. S1b). This relationship demonstrates that differences in plant community composition increase with geographic distance in the floodplain habitat.

*Forage transects quantify woodland plant communities*
Because understory plot data only existed for the floodplain habitat, we also evaluated the relationship between plant communities and distance using data from forage transects (N = 100) sampled in May–June 2019 as part of a separate study (Walker, unpublished), most of which fell within the woodland (Fig. 1a). The survey was designed to evaluate the distribution of key forages species ($n$ = 32)—those that accounted for >95% of the diet of bushbuck and other spiral horned antelopes (*Tragelaphus* spp.)—previously quantified via DNA metabarcoding in Gorongosa (Pansu et al. 2019). Forage transect locations were randomly distributed throughout the main road network in our study area (Fig. S1a) and assigned a random orientation (0-360°).

Along each 100-m transect, surveyors identified the plant rooted every 2.5-m interval (*n* = 40 points per transect) and recorded: (*i*) the plant taxon, if it was a forge species, (ii) 'other', if the taxon was not a forage species, or (*iii*) 'bare', if no plant was rooted at the point.

We quantified dissimilarity of plant community composition (presence/absence of each forage species along each transect) among transects using the Jaccard index. We used a Mantel test (Pansu et al. 2019) to evaluate the relationship between dissimilarity of plant community composition (Jaccard distance) along transects and the pairwise geographic distance between forage transect origins. We found a significant, positive relationship between plant community dissimilarity and geographic distance between forage transects (Fig. S1c). These results demonstrate that differences in plant community composition increase with geographic distance in the woodland habitat.

*Vegetation structure*
Plant species diversity is correlated with vegetation structure (Simonson et al. 2012, Davies and Asner 2014, Guo et al. 2017, George-Chacon et al. 2019). We thus used vegetation structure as a proxy for plant availability within bushbuck home ranges (95% minimum convex polygon, MCP). Methods for quantifying vegetation structure within bushbuck home ranges are outlined in the Main Text. We quantified the pairwise difference in vegetation structure between bushbuck home ranges using the Bray-Curtis index. We then evaluated whether differences in vegetation structure (Bray-Curtis distance) increased with pairwise geographic distance between bushbuck home range centroids (the arithmetic mean position of GPS fixes from each individual). Consistent with results from more direct measures of plant community composition, we found a significant, positive relationship between differences in vegetation structure and geographic distance between home range centroids (Fig. S1d). These results support our assumption that geographic distance between bushbuck home ranges is a suitable proxy for differences in plant community compositions.

*Termite mound density as a proxy for plant communities*
We used termite mound density as a proxy for plant community composition within bushbuck home ranges based on previous work in Gorongosa (Daskin et al. 2022) and elsewhere in southern Africa (Tinley 1977, Davies et al. 2015) documenting significant differences between mound-affiliated plant communities and plant communities in surrounding matrix habitats. Termite mounds are distributed throughout the Rift Valley floor in Gorongosa (Fig.1b) and are pervasive in the woodland habitat (Appendix 1: Fig.S1a) (Tinley 1977). Termite mounds are created by fungus farming termites (Macrotermitinae) that accumulate large mounds of earth and concentrate nutrients and moisture in the soils around their mounds, leading to distinctive plant communities associated with mounds that are more productive than plants in the surrounding matrix habitat (Grant and Scholes 2006). Thus, termite mounds are considered 'foraging hotspots' for large herbivores, which take advantage of the relatively high-quality plants associated with the nutrient and moisture-rich soils on and around the mound (Levick et al 2010). We used digital terrain models derived from LiDAR data collected in 2019 to map the distribution of termite mounds across our study area and quantify the density of mounds within bushbuck home ranges. Using the hillshade tool in ArcGIS to visually transform the digital terrain model, we manually digitized the locations of termite mounds using changes in slope and shape to delineate mounds. Although we did not directly assess the accuracy of this approach, a

previous study that used automated classification to map termite mounds in similar habitat from LiDAR data with coarser resolution (1.12 m) detected 78-90% of mounds >0.5-m tall (Davies et al. 2014), which are those most likely to be meaningful to antelopes. We used a Mantel test (Pansu et al. 2019) to evaluate the relationship between the pairwise difference in mound density within bushbuck home ranges (95% MCP) and the pairwise geographic distance between home range centroids. We found a significant, positive relationship between pairwise difference in mound density within bushbuck home ranges and geographic distance between home ranges (Fig. S1e). These results demonstrate that differences in the availability of mound-associated forage resources increase with geographic distance.

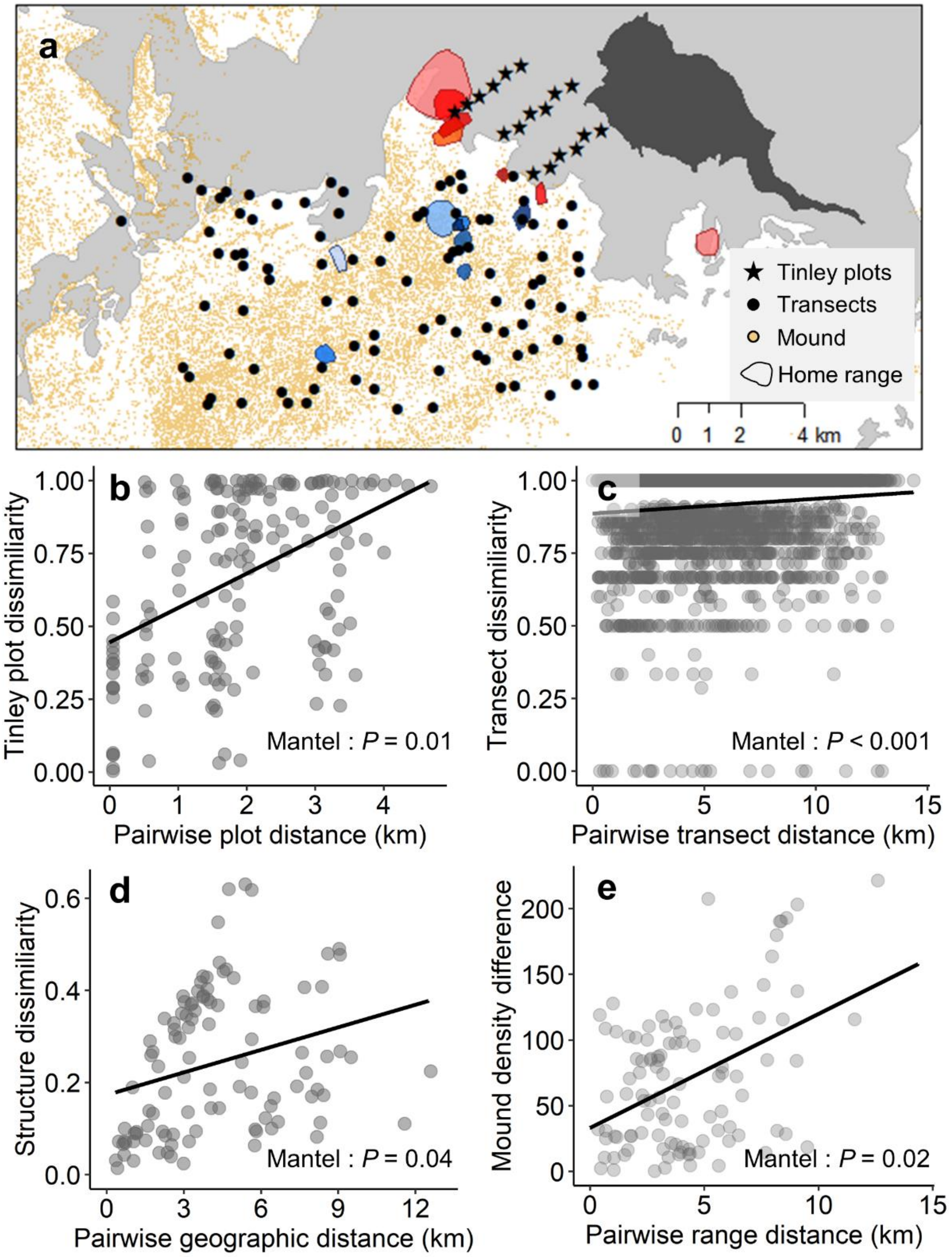


**Figure S1.** Plant community dissimilarity increases with geographic distance in Gorongosa National Park, Mozambique. (A) Map of study area illustrating the locations of previous vegetation surveys (Tinley plots, stars; transects, circles), termite mounds (tan dots), and bushbuck home-range locations relative to floodplain (light grey) and woodland (white) habitats surrounding Lake Urema (dark grey). Bushbuck home ranges (95% minimum convex polygons) affiliated with the floodplain are noted in shades of red and those associated with the woodland are noted in shades of blue. P-values in each panel are from Mantel's permutation tests for similarity between two matrices.

**Literature Cited:**


Braun-Blanquet, J. 1932. *Plant sociology: Study of plant communities*. New York, NY: McGraw-Hill.

Daskin, J. H., J. A. Becker, T. R. Kartzinel, A. B. Potter, R. H. Walker, F. A. A. Ericksson, C. Buoncore, A. Getraer, R. A. Long, and R. M. Pringle. 2022. Allometry of behavior and niche differentiation among congeneric African antelopes. Ecological Monographs (in press).

Davies, A. B., and G. P. Asner. 2014. Advances in animal ecology from 3D-LiDAR ecosystem mapping. Trends in Ecology and Evolution 29: 681-691.

Davies, A. B., S. R. Levick, G. P. Asner, M. P. Robertson, B. J. van Rensburg, and C. L. Parr. 2014. Spatial variability and determinates of termite mounds throughout a savanna catchment. Ecography 37: 1-11.

Davies, A. B., C. A. Baldeck, and G. P. Asner. 2015. Termite mounds alter the spatial distribution of African savanna tree species. Journal of Biogeography 43: 301-313.

George-Chacon, S. P., J. M. Dupuy, A. Peduzzi, and J. L. Hernandez-Stefanoni. 2019. Combining high resolution satellite imagery and lidar data to model woody species diversity of tropical dry forests. Ecological Indicators 101: 975-984.

Grant, C. and M. Scholes. 2006. The importance of nutrient hot-spots in the conservation and management of large wild mammalian herbivores in semi-arid savannas. Biological Conservation: 130, 426-437.

Guo, X., N. C. Coops, P. Tompalski, S. E. Nielsen, C. W. Bater, and J. J. Stadt. 2017. Regional mapping of vegetation structure for biodiversity monitoring using airborne lidar data. Ecological Informatics 38: 50-61.

Guyton, J.A., J. Pansu, M.C. Hutchinson, T.R. Kartzinel, A.B. Potter, T.C. Coverdale, J.H. Daskin, A.G. da Conceição, M.J. Peel, M.E. Stalmans, and R.M. Pringle. 2020. Trophic rewilding revives biotic resistance to shrub invasion. Nature Ecology & Evolution 4:712-724.

Levick, S. R., G. P. Asner, T. Kennedy-Bowdoin, and D. E. Knapp. 2010. The spatial extent of termite influences on herbivore browsing in an African savanna, Biological Conservation 143: 2462-2467.

Pansu, J, J.A. Guyton, A.B. Potter, J.L. Atkins, J.H. Daskin, B. Wursten, T.R. Kartzinel, and R.M. Pringle. 2019. Trophic ecology of large herbivores in a reassembling African ecosystem. Journal of Ecology 107: 1355-1376.

Simonson, W. D., H. D. Allen, and D. A. Coomes. 2012. Use of airborne lidar system to model plant species composition and diversity of Mediterranean oak forests. Conservation Biology 26: 840-850.

Tinley, K. L. 1977. *Framework of the Gorongosa ecosystem, Mozambique*. Ph.D. thesis, University of Pretoria.

Tobler, W. 1970. A computer movie simulating urban growth in the Detroit region. Economic Geography **46**:234-240.

Walker, R.H., M.C. Hutchinson, A.B. Potter, J.A. Becker, R. A. Long, and R.M. Pringle. 2022. Mechanisms of individual variation in large herbivore diets: roles of spatial heterogeneity and state-dependent foraging, Dryad, Dataset, https://doi.org/10.5061/dryad.crjdfn364.
