# Supplementary material for: Mechanisms of individual variation in large herbivore diets: Roles of spatial heterogeneity and state‐dependent foraging
Source: Ecology. 2023 Jan 3;104(2):e3921. doi: 10.1002/ecy.3921 (PMC10078531; doi:10.1002/ecy.3921)
Supplement: Supplementary file 5 — Appendix S5. [file ECY-104-0-s003.pdf]

Supporting information. Walker, R. H., M. C. Hutchinson, A.B. Potter, J. A. Becker, R. A. Long, and R. M. Pringle. 2022. **Mechanisms of individual variation in large herbivore diets: roles of spatial heterogeneity and state-dependent foraging.** *Ecology*.

Appendix S5. Supplementary results illustrating the relationship between nutritional condition and intensity of use index calculated with all hourly GPS data available within the sampling period.

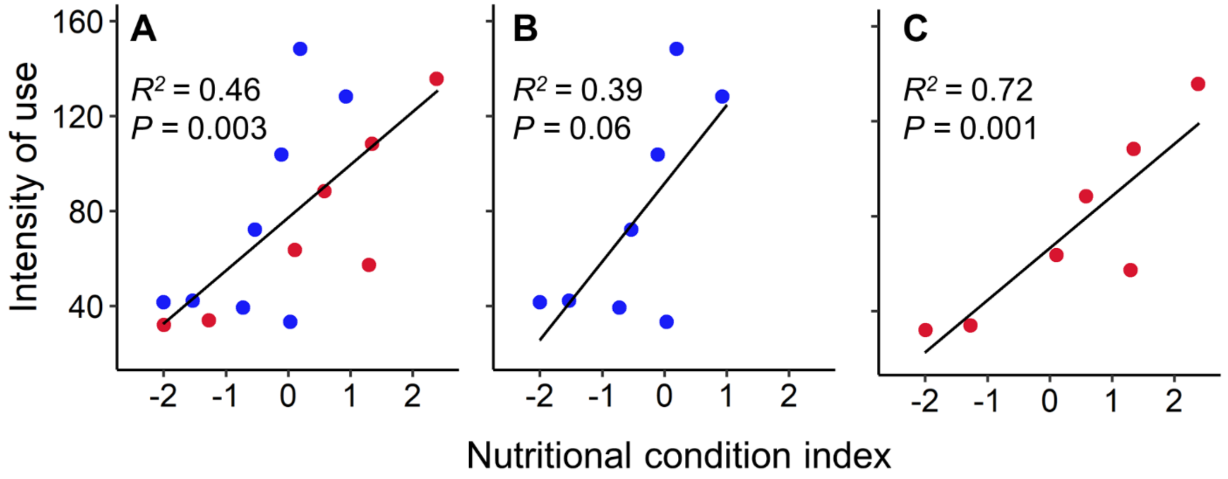

Figure S1. Relationship between nutritional condition and intensity of use index calculated using all available hourly GPS data collected during bushbuck sampling periods (June – August) in Gorongosa National Park, Mozambique. Blue points represent woodland-associated bushbuck; red points represent floodplain-associated bushbuck. Consistent with our findings from data rarified to the first 21 days after capture (the period for which hourly data were available for all sampled individuals), we found a strong, positive relationship between nutritional condition and intensity of home-range use for the population as a whole (A) and for bushbuck associated with the floodplain habitat (C), and a marginally significant relationship for woodland-associated bushbuck (B). Although results are consistent between the two methods of calculating the index, intensity of use is sensitive to sample size (Almeida et al. 2019), and thus we present only results using the rarified dataset in the Main Text (Fig. 4d,e,f).

Literature Cited

Almeida, P.J., M.V. Viera, M. Kajin, G. Forero-Medina, and R. Cerqueira. 2010. Indices of movement behavior: conceptual background, effects of scale and location errors. Zoologia, **27**: 674-680.
