# Supplementary material for: Mechanisms of individual variation in large herbivore diets: Roles of spatial heterogeneity and state‐dependent foraging
Source: Ecology. 2023 Jan 3;104(2):e3921. doi: 10.1002/ecy.3921 (PMC10078531; doi:10.1002/ecy.3921)
Supplement: Supplementary file 6 — Appendix S6. [file ECY-104-0-s002.pdf]

Supporting information. Walker, R. H., M. C. Hutchinson, A.B. Potter, J. A. Becker, R. A. Long, and R. M. Pringle. 2022. **Mechanisms of individual variation in large herbivore diets: roles of spatial heterogeneity and state-dependent foraging.** *Ecology*.

Appendix S6: Supplementary diet analyses based on occurrence (presence/absence) data.

Table S1. Pairwise contrasts in diet based on occurrence (presence/absence) data each pair of bushbuck in the study population using permutational multivariate analysis of variance (*n* = 9,999 permutations) and the Holm-Bonferroni method to control for familywise error rate (‘Adjusted *P*’). These results parallel those presented in Appendix S4: Table S2 using relative read abundance diet data. ‘Pairwise comparison’ identifies the individual diets represented in each contrast. ‘Type’ describes the habitat affiliation of each bushbuck in the contrast (‘W’ indicates a bushbuck affiliated with woodland habitat; ‘FP’ indicated affiliation with the floodplain). Contrasts that were not statistically significant ($P > 0.05$) after accounting for familywise error rate are noted with ‘n.s.’

Pairwise comparison	Type	DF	pseudo-*F*	R^2	*P*	Adjusted *P*
32005_19 vs 31473_19	W v W	1,20	22.56	0.52	0.0001	0.011
31473_18 vs 31473_19	W v W	1,19	22.44	0.54	0.0001	0.011
32005_19 vs 32007_19	W v W	1,20	20.19	0.50	0.0001	0.011
32005_19 vs 32009_18	W v W	1,24	18.07	0.43	0.0001	0.011
32005_19 vs 32010_19	W v W	1,19	45.73	0.70	0.0001	0.011
32005_19 vs 31471_18	W v W	1,20	22.65	0.53	0.0001	0.011
32005_19 vs 31472_18	W v W	1,22	25.38	0.54	0.0001	0.011
32005_19 vs 31473_18	W v W	1,21	22.56	0.52	0.0001	0.011
32007_19 vs 32009_18	W v W	1,22	24.85	0.53	0.0001	0.011
32007_19 vs 32010_19	W v W	1,17	9.73	0.36	0.0001	0.011
32007_19 vs 31471_18	W v W	1,18	13.48	0.43	0.0001	0.011
32007_19 vs 31472_18	W v W	1,20	19.41	0.49	0.0001	0.011
32007_19 vs 31473_18	W v W	1,19	18.32	0.49	0.0001	0.011
32007_19 vs 31473_19	W v W	1,18	10.97	0.38	0.0002	0.011
32009_18 vs 32010_19	W v W	1,21	7.90	0.27	0.0001	0.011
32009_18 vs 31471_18	W v W	1,22	19.95	0.48	0.0001	0.011
32009_18 vs 31472_18	W v W	1,24	11.32	0.32	0.0001	0.011
32009_18 vs 31473_18	W v W	1,23	10.16	0.31	0.0001	0.011
32009_18 vs 31473_19	W v W	1,22	25.56	0.54	0.0001	0.011
32010_19 vs 31471_18	W v W	1,17	8.08	0.32	0.0001	0.011
32010_19 vs 31472_18	W v W	1,19	6.67	0.26	0.0001	0.011

32010_19 vs 31473_18	W v W	1,18	8.60	0.32	0.0001	0.011	
32010_19 vs 31473_19	W v W	1,17	11.35	0.40	0.0001	0.011	
31471_18 vs 31472_18	W v W	1,20	5.89	0.23	0.0002	0.011	
31471_18 vs 31473_18	W v W	1,19	5.45	0.22	0.0003	0.011	
31471_18 vs 31473_19	W v W	1,18	16.21	0.47	0.0002	0.011	
31472_18 vs 31473_18	W v W	1,21	2.86	0.12	0.0187	0.056	n.s.
31472_18 vs 31473_19	W v W	1,20	26.10	0.57	0.0001	0.011	
32006_18 vs 31765_18	FP v FP	1,16	7.40	0.32	0.0011	0.011	
32006_18 vs 32010_18	FP v FP	1,20	4.09	0.17	0.0033	0.013	
32006_18 vs 32012_18	FP v FP	1,23	14.03	0.38	0.0001	0.011	
32006_18 vs 31471_19	FP v FP	1,22	8.42	0.28	0.0001	0.011	
32006_18 vs 31472_19	FP v FP	1,22	14.45	0.40	0.0001	0.011	
32006_18 vs 31483_18	FP v FP	1,16	2.24	0.12	0.0577	0.115	n.s.
32010_18 vs 32012_18	FP v FP	1,21	7.64	0.27	0.0001	0.011	
32010_18 vs 31765_18	FP v FP	1,14	6.22	0.31	0.0004	0.011	
32010_18 vs 31471_19	FP v FP	1,20	6.62	0.25	0.0001	0.011	
32010_18 vs 31472_19	FP v FP	1,20	9.98	0.33	0.0001	0.011	
32010_18 vs 31483_18	FP v FP	1,14	1.57	0.10	0.1587	0.159	n.s.
32012_18 vs 31765_18	FP v FP	1,17	14.07	0.45	0.0003	0.011	
32012_18 vs 31471_19	FP v FP	1,23	8.43	0.27	0.0001	0.011	
32012_18 vs 31472_19	FP v FP	1,23	18.00	0.44	0.0001	0.011	
32012_18 vs 31483_18	FP v FP	1,17	10.47	0.38	0.0002	0.011	
31765_18 vs 31471_19	FP v FP	1,16	7.58	0.32	0.0001	0.011	
31765_18 vs 31472_19	FP v FP	1,16	8.27	0.34	0.0002	0.011	
31765_18 vs 31483_18	FP v FP	1,11	4.38	0.30	0.0024	0.012	
31471_19 vs 31472_19	FP v FP	1,22	4.22	0.16	0.0005	0.011	
31471_19 vs 31483_18	FP v FP	1,16	33.33	0.61	0.0001	0.011	
31472_19 vs 31483_18	FP v FP	1,16	7.47	0.32	0.0004	0.011	
32005_19 vs 32012_18	W v FP	1,23	57.10	0.71	0.0001	0.011	
32005_19 vs 32006_18	W v FP	1,22	48.29	0.69	0.0001	0.011	
32005_19 vs 32010_18	W v FP	1,20	45.73	0.70	0.0001	0.011	
32005_19 vs 31765_18	W v FP	1,16	26.28	0.62	0.0002	0.011	
32005_19 vs 31471_19	W v FP	1,22	21.53	0.49	0.0001	0.011	
32005_19 vs 31472_19	W v FP	1,22	27.46	0.56	0.0001	0.011	
32005_19 vs 31483_18	W v FP	1,16	31.99	0.67	0.0002	0.011	
32007_19 vs 32010_18	W v FP	1,18	64.52	0.78	0.0001	0.011	

32007_19 vs 32012_18	W v FP	1,21	68.56	0.77	0.0001	0.011
32007_19 vs 31765_18	W v FP	1,14	42.96	0.75	0.0004	0.011
32007_19 vs 31471_19	W v FP	1,20	30.91	0.61	0.0001	0.011
32007_19 vs 31472_19	W v FP	1,20	40.75	0.67	0.0001	0.011
32007_19 vs 31483_18	W v FP	1,14	46.50	0.77	0.0005	0.011
32009_18 vs 32010_18	W v FP	1,22	56.12	0.72	0.0001	0.011
32009_18 vs 32012_18	W v FP	1,25	71.78	0.74	0.0001	0.011
32009_18 vs 31765_18	W v FP	1,18	28.19	0.61	0.0001	0.011
32009_18 vs 31471_19	W v FP	1,24	25.52	0.52	0.0001	0.011
32009_18 vs 31472_19	W v FP	1,24	32.81	0.58	0.0001	0.011
32009_18 vs 31483_18	W v FP	1,18	38.08	0.68	0.0001	0.011
32010_19 vs 32012_18	W v FP	1,20	44.65	0.69	0.0001	0.011
32010_19 vs 31765_18	W v FP	1,13	25.76	0.66	0.0004	0.011
32010_19 vs 31471_19	W v FP	1,19	20.62	0.52	0.0001	0.011
32010_19 vs 31472_19	W v FP	1,19	27.16	0.59	0.0001	0.011
32010_19 vs 31483_18	W v FP	1,13	26.07	0.67	0.0004	0.011
31471_18 vs 31471_19	W v FP	1,21	31.28	0.61	0.0001	0.011
31471_18 vs 31472_19	W v FP	1,20	44.20	0.69	0.0001	0.011
31471_18 vs 31483_18	W v FP	1,14	48.09	0.77	0.0005	0.011
31472_18 vs 31472_19	W v FP	1,22	46.49	0.68	0.0001	0.011
31472_18 vs 31483_18	W v FP	1,16	56.18	0.78	0.0004	0.011
31473_18 vs 31483_18	W v FP	1,15	39.75	0.73	0.0002	0.011
31473_19 vs 31483_18	W v FP	1,14	42.95	0.75	0.0002	0.011
32006_18 vs 32007_19	FP v W	1,20	63.14	0.76	0.0001	0.011
32006_18 vs 32009_18	FP v W	1,24	52.47	0.69	0.0001	0.011
32006_18 vs 32010_19	FP v W	1,19	40.44	0.68	0.0001	0.011
32006_18 vs 31471_18	FP v W	1,20	62.18	0.76	0.0001	0.011
32006_18 vs 31472_18	FP v W	1,22	71.07	0.76	0.0001	0.011
32006_18 vs 31473_18	FP v W	1,21	53.59	0.72	0.0001	0.011
32006_18 vs 31473_19	FP v W	1,20	58.25	0.74	0.0001	0.011
32010_18 vs 32010_19	FP v W	1,17	39.37	0.70	0.0001	0.011
32010_18 vs 31471_18	FP v W	1,18	64.72	0.78	0.0001	0.011
32010_18 vs 31472_18	FP v W	1,20	76.95	0.79	0.0001	0.011
32010_18 vs 31473_18	FP v W	1,19	54.77	0.74	0.0001	0.011
32010_18 vs 31473_19	FP v W	1,18	59.53	0.77	0.0001	0.011
32012_18 vs 31471_18	FP v W	1,21	71.01	0.77	0.0001	0.011

32012_18 vs 31472_18	FP v W	1,23	85.25	0.79	0.0001	0.011
32012_18 vs 31473_18	FP v W	1,22	66.21	0.75	0.0001	0.011
32012_18 vs 31473_19	FP v W	1,21	65.16	0.76	0.0001	0.011
31765_18 vs 31471_18	FP v W	1,14	45.90	0.77	0.0002	0.011
31765_18 vs 31472_18	FP v W	1,16	47.13	0.75	0.0001	0.011
31765_18 vs 31473_18	FP v W	1,15	34.26	0.70	0.0001	0.011
31765_18 vs 31473_19	FP v W	1,14	39.49	0.74	0.0001	0.011
31471_19 vs 31472_18	FP v W	1,22	33.86	0.61	0.0001	0.011
31471_19 vs 31473_18	FP v W	1,21	24.37	0.54	0.0001	0.011
31471_19 vs 31473_19	FP v W	1,20	29.01	0.59	0.0001	0.011
31472_19 vs 31473_18	FP v W	1,21	33.33	0.61	0.0001	0.011
31472_19 vs 31473_19	FP v W	1,20	37.71	0.65	0.0001	0.011

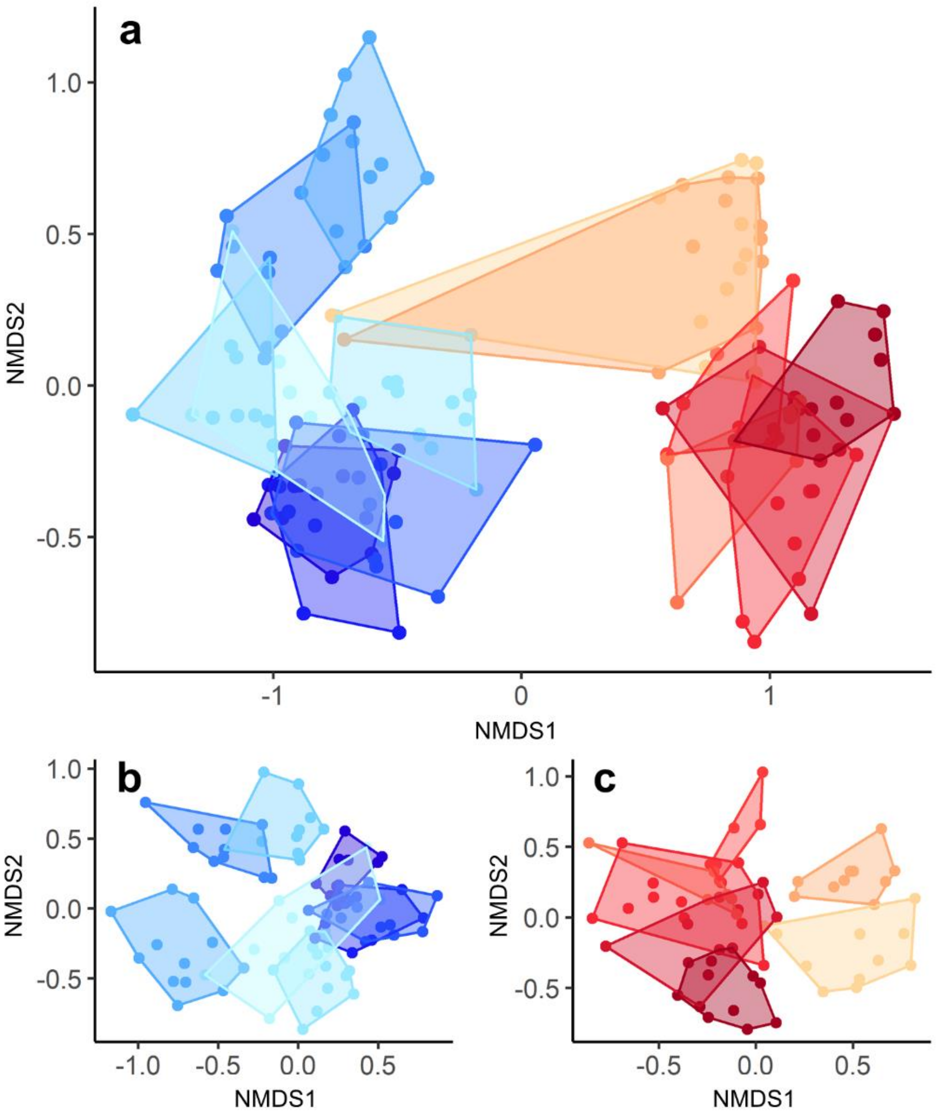

Figure S1. Non-metric multidimensional scaling (NMDS) ordinations showing relative dissimilarity (Jaccard index) in the taxonomic composition (presence/absence) of individual fecal samples (points; *n* = 160) and dry-season diets (polygons; *n* = 15) of bushbuck in Gorongosa National Park, Mozambique. This occurrence-based analysis of all diet plant taxa accounting for > 1% of reads in fecal DNA metabarcoding analysis parallels the analysis of relative read abundance presented in Figure 2. Results for the full population are shown in A, and results partitioned by habitat association are shown in B (woodland, blue) and C (floodplain, red). Points in closer proximity to one another indicate more similar diets; polygons are convex hulls around all samples from each individual. Two individuals captured in woodland moved into floodplain shortly after collaring (outlying red points in A are those collected at capture); we excluded these initial woodland samples from the analysis of floodplain diet (C). We observed significant differences in individual diets, both across all individuals (perMANOVA: pseudo-$F_{14,144}$ = 28.74, , $P \leq 0.001$, R^2 = 0.74) and within habitat types (woodland: pseudo-$F_{7,80}$= 15.15, $P \leq 0.001$, R^2 = 0.57; floodplain: psudeo-$F_{6,62}$ = 11.27, $P \leq 0.001$, R^2= 0.52).

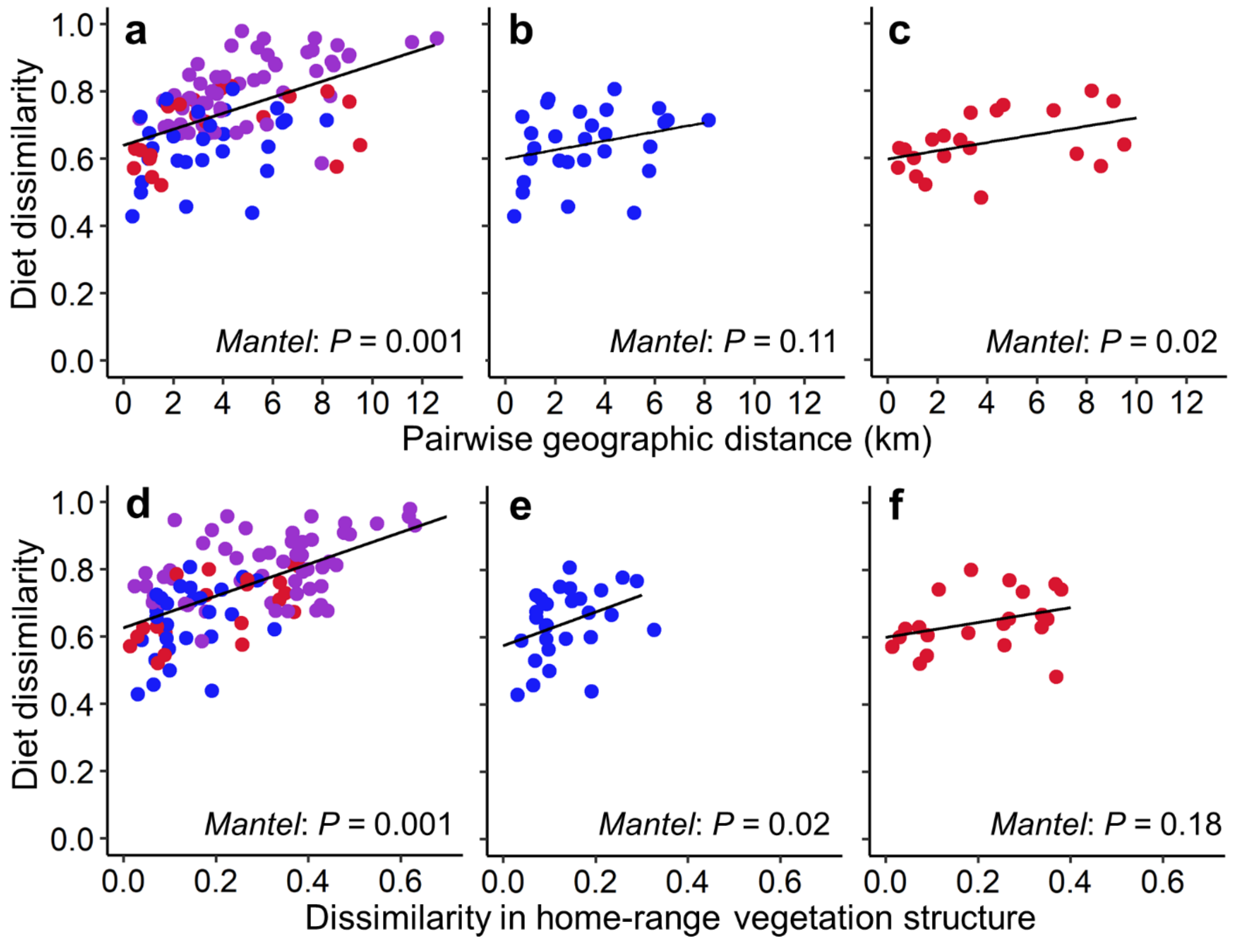

Figure S2. Relationship between bushbuck diet composition (presence/absence) and spatial variation in the distribution of resources in Gorongosa. This occurrence-based analysis of all diet plant taxa accounting for > 1% of reads in fecal DNA metabarcoding analysis parallels the analysis of relative read abundance presented in Figure 3. We evaluated the relationship between pairwise diet dissimilarity (Jaccard index) and distance between home-range centroids (km) for all pairs of GPS-collared bushbuck (A), woodland-associated bushbuck (B), and floodplain-associated bushbuck (C). Additionally, we evaluated the relationship between pairwise diet dissimilarity and dissimilarity in home-range vegetation structure (Bray-Curtis index) between all pairs of GPS-collared bushbuck (D), woodland-associated bushbuck (E), and floodplain-associated bushbuck (F). Blue points illustrate pairwise comparison between two woodland-affiliated individuals, red points between two floodplain-affiliated individuals, purple points between a woodland and a floodplain affiliated individual. We quantified vegetation structure by calculating the proportion of LiDAR points classified as ground-level, low, medium, and high vegetation and using the Bray-Curtis index to quantify pairwise compositional dissimilarity between home ranges based on those proportions. P-values in each panel are from Mantel's permutation tests for similarity between two matrices.

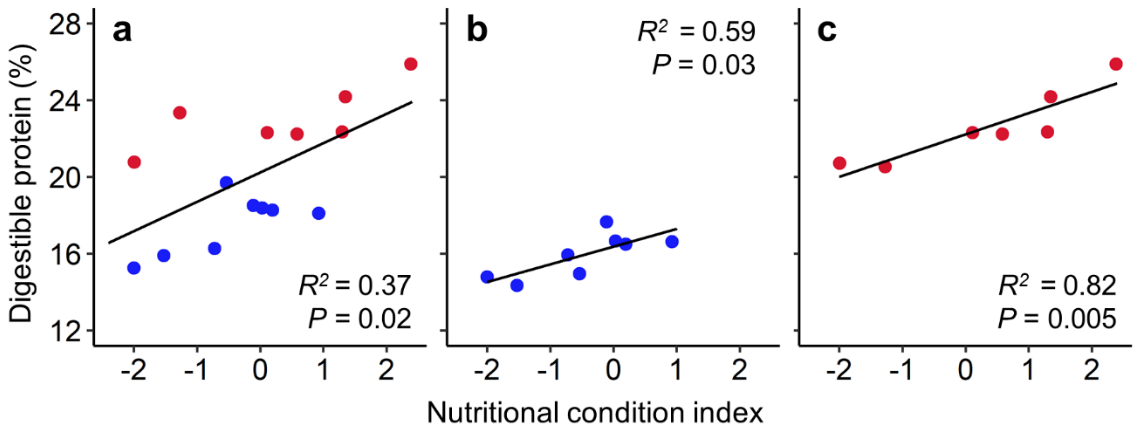

Figure S3. Relationships between a multivariate index of nutritional condition (see Methods) and bushbuck diet quality quantified with occurrence (presence/absence) data in Gorongosa. We estimated the quality of individual bushbuck diets by calculating weighted average of DP in the standardized diet of each bushbuck assuming equal contribution of each present mOTU to the bushbuck diet as the weighting factor. This occurrence-based analysis of all diet plant taxa accounting for > 1% of reads in fecal DNA metabarcoding analysis parallels the analysis of relative read abundance presented in Figure 4d,e,f. Blue points represent bushbuck with home ranges affiliated with woodland habitat; red points represent bushbuck with home ranges affiliated with floodplain habitat. R^2 and P-values are from ordinary least-squares linear regression models.

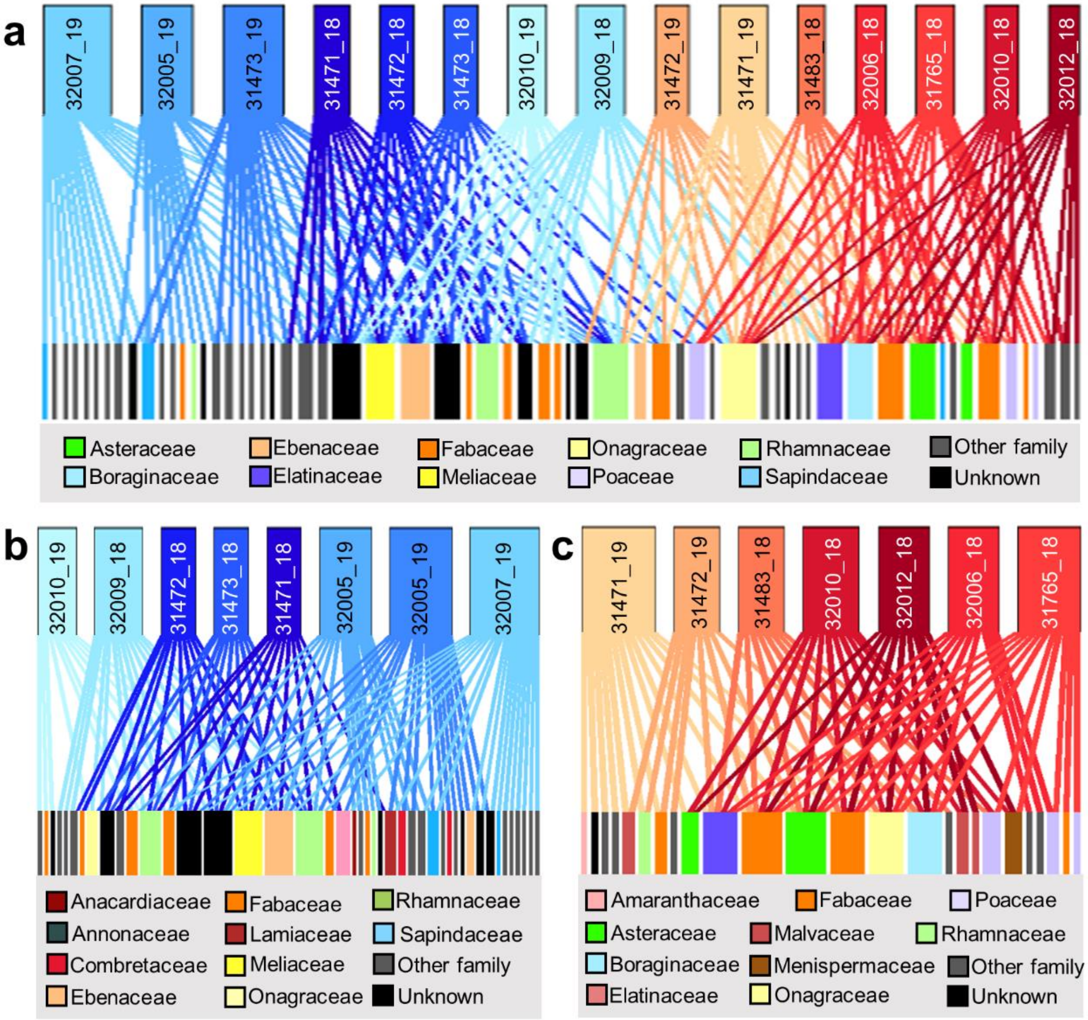

Figure S4. Bipartite plant-herbivore interaction network for individual bushbuck calculated from presence/absence data. This occurrence-based analysis of all diet plant taxa accounting for > 1% of reads in fecal DNA metabarcoding analysis parallels the relative read abundance-based results presented in Appendix 4: Figure S1. Results for all bushbuck in the population are shown in A and results partitioned by habitat association are shown in B (woodland) and C (floodplain). Lines connect individuals (top) to dietary plant mOTUs (bottom, colored by plant family). Width of the top rectangles represent the relative breadth of individual diets; width of the bottom rectangles represent the relative abundance of each plant mOTU in the database.
