# Supplementary material for: Mechanisms of individual variation in large herbivore diets: Roles of spatial heterogeneity and state‐dependent foraging
Source: Ecology. 2023 Jan 3;104(2):e3921. doi: 10.1002/ecy.3921 (PMC10078531; doi:10.1002/ecy.3921)
Supplement: Supplementary file 7 — Appendix S7. [file ECY-104-0-s006.pdf]

Supporting information. Walker, R. H., M. C. Hutchinson, A.B. Potter, J. A. Becker, R. A. Long, and R. M. Pringle. 2022. **Mechanisms of individual variation in large herbivore diets: roles of spatial heterogeneity and state-dependent foraging.** *Ecology.*

Appendix S7. Further exploring relationships among nutritional condition, diet diversity, and home range size.

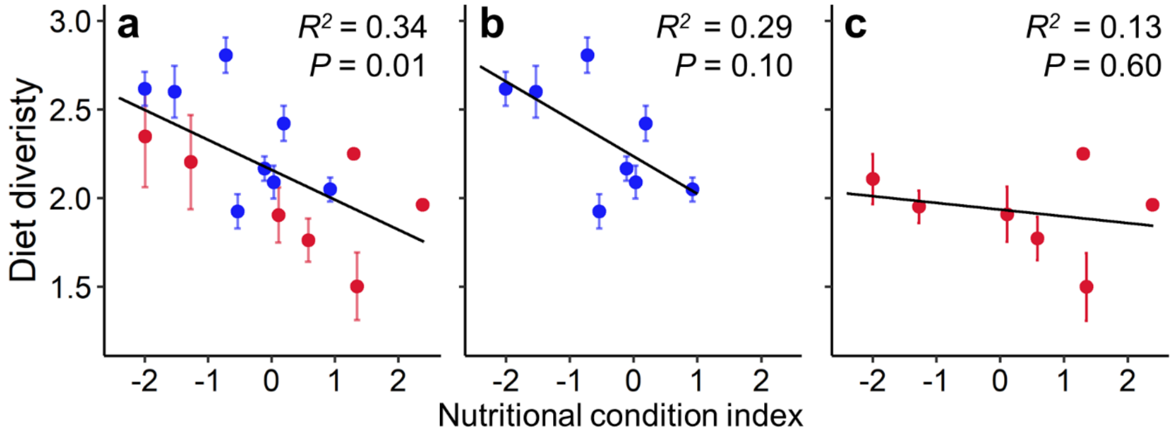

Figure S1. Relationship between bushbuck nutritional condition (multivariate index of condition; see Methods) and diet diversity (Shannon Weaver index) in Gorongosa National Park, Mozambique. Blue points represent woodland-associated bushbuck; red points represent floodplain-associated bushbuck. Bushbuck in good nutritional condition had less diverse diets than their counterparts in poorer condition (A; mean ± SD unique mOTUs across 1,000 iterations of randomly drawing 6 samples for each individual, see Methods). This relationship persisted for bushbuck associated with woodland habitat (B) but not for bushbuck associated with floodplain habitat (C). R^2 and P-values are reported from ordinary least-squares linear regression models.

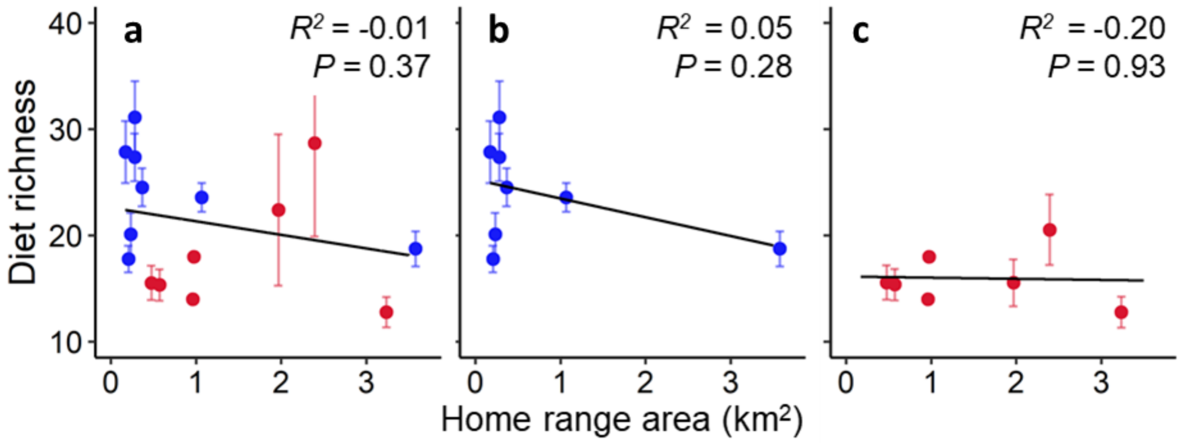

Figure S2. Relationship between bushbuck diet richness and home range area (km^2) in Gorongosa National Park, Mozambique. Blue points represent woodland-associated bushbuck; red points represent floodplain-associated bushbuck. We used 95% minimum convex polygons (MCP) derived from hourly GPS-location data to estimate individual bushbuck home ranges during our sampling period in each year. We observed no relationship between diet richness (mean ± *SD* mOTUs among 1,000 iterations of randomly drawing 6 samples for each individual) and home-range size across (A) or within (B,C) habitat types. R^2 and P-values are reported from ordinary least-squares linear regression models.
